# Fungicide-free management of Alternaria leaf blotch and fruit spot on apple indicates *Alternaria spp.* as secondary colonizer

**DOI:** 10.1038/s41598-023-35448-2

**Published:** 2023-05-24

**Authors:** Ulrich E. Prechsl, Werner Rizzoli, Klaus Marschall, E. R. Jasper Wubs

**Affiliations:** 1 Research Centre Laimburg, Laimburg 6, 39040 Auer/Ora, BZ Italy; 2 Sustainable Agroecosystems Group, Institute of Agricultural Sciences, Department of Environmental Systems Science, ETH Zürich, Universitätstrasse 2, 8092 Zürich, Switzerland; 3 Department of Terrestrial Ecology, Netherlands Institute of Ecology (NIOO-KNAW), P.O. Box 50, 6700 AB Wageningen, The Netherlands; 4 Present Address: Terra Institute, Säbenertorgasse 2, 39042 Brixen, BZ Italy

**Keywords:** Fungi, Pathogens

## Abstract

The fungal genus *Alternaria* is a pan-global pathogen of > 100 crops, and is associated with the globally expanding Alternaria leaf blotch in apple (*Malus x domestica* Borkh.) which leads to severe leaf necrosis, premature defoliation, and large economic losses. Up to date, the epidemiology of many *Alternaria* species is still not resolved as they can be saprophytic, parasitic or shift between both lifestyles and are also classified as primary pathogen able to infect healthy tissue. We argue that *Alternaria spp.* does not act as primary pathogen, but only as a necrosis-dependent opportunist. We studied the infection biology of *Alternaria spp.* under controlled conditions and monitored disease prevalence in real orchards and validated our ideas by applying fungicide-free treatments in 3-years field experiments. *Alternaria spp.* isolates were not able to induce necroses in healthy tissue, but only when prior induced damages existed. Next, leaf-applied fertilizers, without fungicidal effect, reduced Alternaria-associated symptoms (− 72.7%, SE: ± 2.5%) with the same efficacy as fungicides. Finally, low leaf magnesium, sulphur, and manganese concentrations were consistently linked with Alternaria-associated leaf blotch. Fruit spot incidence correlated positively with leaf blotch, was also reduced by fertilizer treatments, and did not expand during storage unlike other fungus-mediated diseases. Our findings suggest that *Alternaria spp.* may be a consequence of leaf blotch rather than its primary cause, as it appears to colonize the physiologically induced leaf blotch. Taking into account existing observations that Alternaria infection is connected to weakened hosts, the distinction may appear slight, but is of great significance, as we can now (a) explain the mechanism of how different stresses result in colonization with *Alternaria spp.* and (b) substitute fungicides for a basic leaf fertilizer. Therefore, our findings can result in significant decreases in environmental costs due to reduced fungicide use, especially if the same mechanism applies to other crops.

## Introduction

The pan-global genus *Alternaria* comprises saprotrophic fungi which can become pathogenic for various crops, especially when host plants are weakened and under stress^1–3^. There are > 100 host plant species, including major crops like potato, tomato, wheat, cabbage, sunflower, cotton, soybean and apple, on which the fungi typically provoke necrotic lesions^4–8^. Due to its wide host range, *Alternaria* is amongst the most damaging pathogens worldwide. On apple (*Malus x domestica* Borkh*)*, Alternaria-associated symptoms include leaf blotch and subsequent fruit spots and can lead to 85% defoliation and up to 80% infested fruits per orchard (Supplementary Fig. 1), leading to vast economic losses^1,7,9–11^. In Asia it is thought to be economically the most important disease for apple production^12,13^. Globally, apple production is dominated by a few varieties: ‘Golden Delicious’, ‘Red Delicious’, ‘Fuji’, ‘Gala’. These top 4 varieties account for 60.3% of global production (when excluding China, for which data quality is less certain, it is 50.8%, data 2015), all of which are susceptible to Alternaria leaf blotch, exacerbating the impact of the disease^14–16^. In addition, it is important to mention that *Alternaria spp.* can cause other diseases in apples, such as moldy core disease, which impacts the fruit's internal quality and results in significant financial losses for apple growers and producers during postharvest storage^10,17,18^.

Since the first report of Alternaria leaf blotch on apple in 1926 (USA), the disease has spread over the apple growing regions across the world (Asia, Russia, Australia), first in regions with arid, warm climate, including Southern Europe^19^, and recently also temperate regions (e.g. Netherlands, 2018)^9,11,14,19–23^. Warm conditions and heat periods are necessary for the development of Alternaria leaf blotch and first symptoms typically occur in early summer (Supplementary Figs. 1a and 2). Subsequent rainfall and drops in temperature in late summer increase the symptoms dramatically (Supplementary Fig. 1b) leading to severe defoliation (Supplementary Fig. 1c) and fruit spots (Supplementary Fig. 1d)^12^. Despite its importance, the epidemiology of *Alternaria* on apple is still poorly understood. Consequently, the plant protection strategy to control Alternaria-disease is not specific and often not effective. Farmers regularly apply fungicides by routine spraying with up to eight treatments per growing season as a harvest insurance strategy^17,24^. Current agricultural policy, however, aims to drastically reduce the use of pesticides (e.g. up to 50% reduction by 2030; European Commission), particularly because synthetic pesticides form one of the most prominent threats to non-target organisms and for contamination of soil and water^25–30^. Thus, there is currently a great demand for alternative approaches in plant protection to safeguard food security.

**Figure 1:**
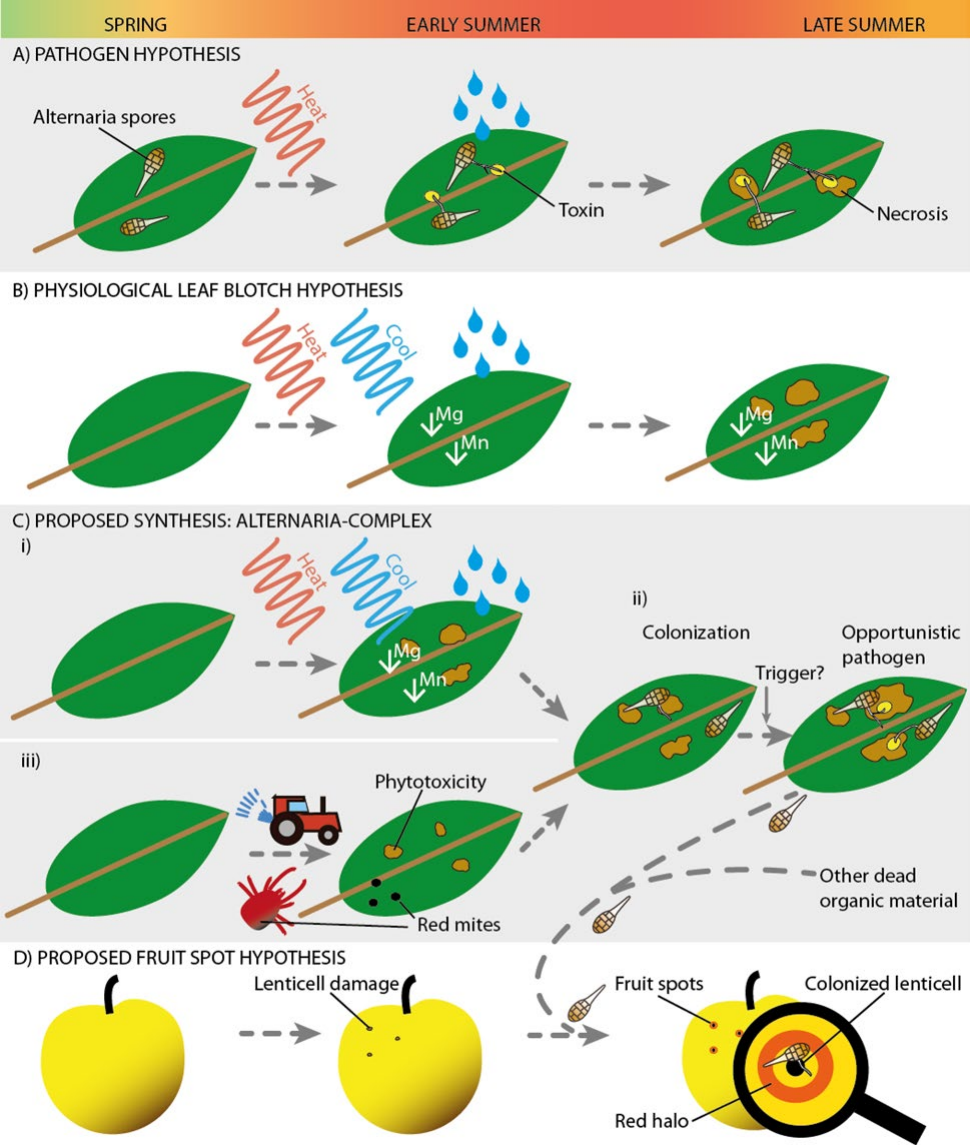
Conceptual model describing existing hypothesis on (Alternaria) leaf and fruit spot formation on apple and our new hypothesis, a synthesis, the Alternaria-complex. There are two existing hypotheses for the development of the same symptoms: (**A**) the pathogen hypothesis for Alternaria leaf blotch (biotic) and (**B**) the physiological leaf blotch hypothesis (abiotic). We propose a synthesis of both (**C**) the Alternaria-complex. Environmental factord lead to nutrient imbalances with formation of leaf spots (**C-i**), which subsequently can be colonized by *Alternaria spp.* (**C-ii**). Other factors such as phytotoxicity of active agents and red mites can also form the necessary conditions for *Alternaria spp.* colonization (**C-iii**). An unknown trigger can shift the saprophytic lifeform to a pathogenic lifeform (toxin production) of *Alternaria spp.*, with the enhancement of the typical symptoms as a consequence. Similarly, for fruit spot formation (**D**). Pre-damaged lenticels are the obligatory precondition for the colonization by *Alternaria spp.*, with the symptoms as a consequence.

Importantly, the symptoms of Alternaria leaf blotch are highly similar to a poorly known physiological disease of apple that was described as ‘physiological (or necrotic) leaf blotch’^13,17,31^. Both diseases are characterized by similar necrotic leaf spots, the same progression of disease and similar climatic conditions favouring disease outbreak (see also the Supplementary "Discussion": Two diseases, same symptoms, Fig. 1a,b). Physiological leaf blotch is thought to be caused by leaf nutrient imbalances, triggered by temperature drops after heat periods in summer^13,31^. Early studies showed that zinc and manganese containing organic fungicides (dithiocarbamates), as well as treatments with magnesium- and manganese-foliar-fertilizers, could reduce the physiological leaf blotch^32^. In contrast, potassium fertilizers, an antagonist for magnesium-uptake, increased the defoliation^33^. However, these previous investigations did not resolve if and what type of nutrient imbalance was responsible for the disease and did not develop an effective cure^31,34^. In addition, fungicides with different modes of action (FRAC code: 3 and P 09) also reduced the physiological leaf blotch, but a pathogen was not identified^35,36^.

In summary, the causes for Alternaria leaf blotch, physiological leaf blotch, and fruit spots are poorly understood. Furthermore, the hypotheses to date are in conflict with the existing evidence: if leaf blotch were purely caused by *Alternaria spp.* infection, then it should not be affected by leaf-applied fertilizers, as they do not have fungicidal activity. Similarly, if nutrient imbalances were the sole cause, then why do fungicides with different modes of action reduce leaf blotch severity? Given the similarity in symptoms, we propose a new synthesis. We hypothesize that the physiological leaf blotch and Alternaria leaf blotch are two interlinked diseases: the Alternaria-complex. Initial leaf necrosis, caused by nutrient imbalances in apple trees (as shown in Fig. 1c), is a necessary prerequisite for the subsequent colonization by *Alternaria spp.*, which in turn can exacerbate the severity of the disease.

We predict (1) that there are distinct leaf nutrient signatures associated with the disease, (2) that *Alternaria spp.* cannot infect healthy apple tissues without pre-existing necroses, and (3) that Alternaria-associated diseases can be controlled by preventing initial leaf necroses by leaf fertilizers.

## Results

### Sulphur, magnesium, and manganese drive initial leaf blotch formation

We studied leaf blotch disease development in apple orchards in South Tyrol (Italy) where leaf blotch was increasingly prevalent from 1999 onwards. To reveal which leaf nutrients might cause the initial leaf blotch, we linked leaf nutrient concentrations (N, P, K, Ca, Mg, S, B, Fe, Mn, Cu, and Zn) to leaf blotch severity using Random Forest analysis (for a detailed analysis see Supplementary Results S1, Supplementary Figs. 4–6, Supplementary Table 5).

**Figure 2:**
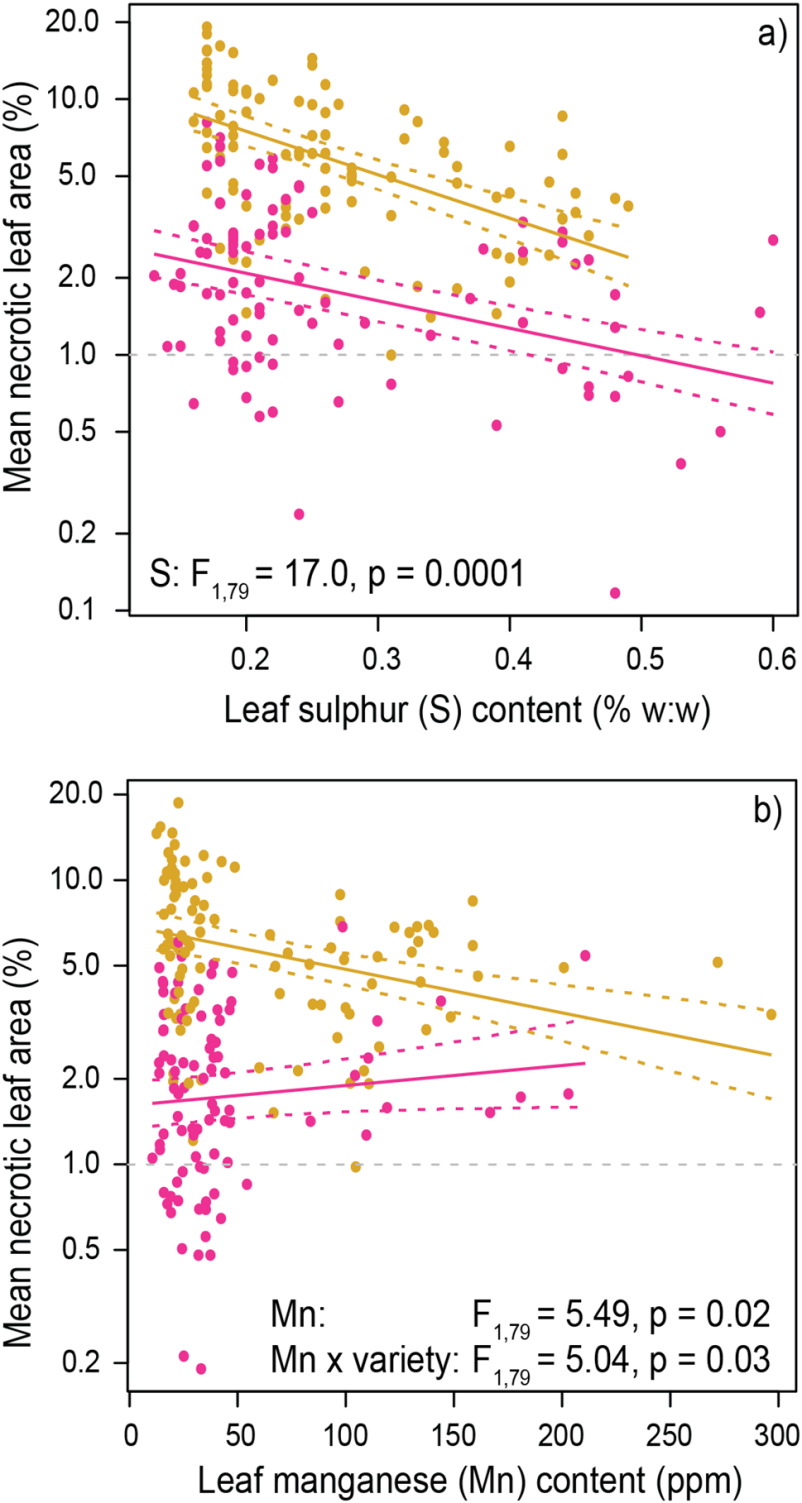
Relationships between leaf blotch severity and leaf nutrient concentrations. A significant relationship was found for sulphur (**a**) and manganese (**b**). Note leaf blotch is plotted on a logarithmic scale. Separate lines were fit for ‘Golden Delicious’ (orange) and ‘Cripps Pink’ (pink) apple varieties. Thick coloured lines show the fitted regression, dotted lines the SEs. The grey dotted lines are shown as a visual aid only. See Supplementary Results S1 for a detailed analysis.

The data was obtained from mature trees of both 'Golden Delicious' and 'Cripps Pink' apple varieties, which are susceptible to *Alternaria spp*., and were used in our field experiment described below. From the predictors, low sulphur concentrations were consistently associated with more severe leaf blotch in both varieties (Fig. 2a). However, sulphur and magnesium were strongly collinear in the dataset (r = 0.78, Supplementary Fig. 4) and we cannot exclude a potential role for magnesium, even though model selection (based on AIC) strongly supported sulphur as the driving element (Supplementary Results S1). In addition, low manganese concentrations were also associated with high leaf blotch severity, but only for ‘Golden Delicious’ (Fig. 2b). To validate these results on independent data, we compared leaf nutrient status in paired ‘Golden Delicious’ orchards in the study area with either high or low leaf blotch incidence in previous years. This analysis showed that high-incidence orchards were indeed associated with low leaf sulphur, magnesium and especially manganese concentrations (Supplementary Results S2, Supplementary Figs. 7 and 8).

### Isolated fungi (*A. alternata sspp. tenuissima & A. alternate sspp. arborescens*) did not infect healthy apple tissue

Next, we studied the infection biology of Alternaria-disease. We tested under controlled conditions if *Alternaria spp.* can infect healthy or injured leaf and fruit tissue. Initially, a leaf disc assay was conducted using a spore suspension (1.25 × 10^6^ spores/ml) of four distinct *Alternaria spp.* isolates (two *A. alternata sspp. tenuissima* and two *A. alternate sspp. arborescens*, as classified by Armitage et al. 2015) that were originally isolated from leaf blotch and fruit spot on apples. However, this assay did not result in leaf necrosis on neither healthy tissue nor on leaf discs manually damaged with pliers (Supplementary Fig. 9; Supplementary Table 7). We found no indications of an infection (yellowing or browning of the leaf epidermis), even though the isolates did develop a dense mycelium on top of the leaf disc surface (Supplementary Fig. 1f). Only when the leaves had been previously attacked and damaged by European red mites (*Panonychus ulmi;* sucking and damaging leaves), did necroses form (Supplementary Fig. S9).

In a second laboratory assay, we artificially inoculated apple fruits with a dense spore suspension of *Alternaria spp.* (*A. alternata sspp. arborescens*; 1.25 × 10^6^ spores/ml), which was originally isolated from infected fruits. After 12 days in the climate chamber (25 °C), we did not detect any fruit spots nor any signs of infection (necrotic spots, mold) (Supplementary Fig. 10). This was also true when apples had fresh, non-necrotic wounds, which should, according to the literature, facilitate an infection^7^. In a third assay, we investigated if fruit spots can expand in number and size during storage (room temperature, ~ 15–20 °C). For this purpose, infected and healthy fruits were stored for two months together in boxes, having surface contact. Here, healthy (control) fruits did not develop any fruit spots, and the number of the fruit spots on infected fruits did not change during storage (Supplementary Fig. 11). Thus, fruit spots did not increase in number and size during storage.

In summary, our study found that under controlled conditions, our Alternaria isolates (*A. alternata sspp. tenuissima* and *A. alternate sspp. arborescens*) were not able to infect healthy tissue or fresh wounds, nor were they able to reproduce the symptoms of leaf blotch and fruit spot in neither leaves nor fruits.

### Alternaria colonizes existing necrosis

To investigate the role of leaf necrosis in *Alternaria spp.* occurrence, we conducted a field experiment in July 2019 on ‘Golden Delicious’ trees. We induced leaf spots using a herbicide (Carfentrazone-ethyl) and examined whether this led to the appearance of *Alternaria spp*. In September, the density of Alternaria-spores was on average 14 times higher on the herbicide-induced leaf spots than on the naturally occurring Alternaria leaf blotch (Supplementary Fig. 12). In October, the spore density had increased on natural Alternaria leaf blotch, but was still considerably (2.7-fold) lower than on herbicide-induced leaf spots (Supplementary Table 8), indicating that pre-existing necroses are an important pre-condition to colonization and that *Alternaria spp.* subsequently follows the necrosis spread.

### Leaf-applied fertilizers equally effective as fungicides

**Figure 3:**
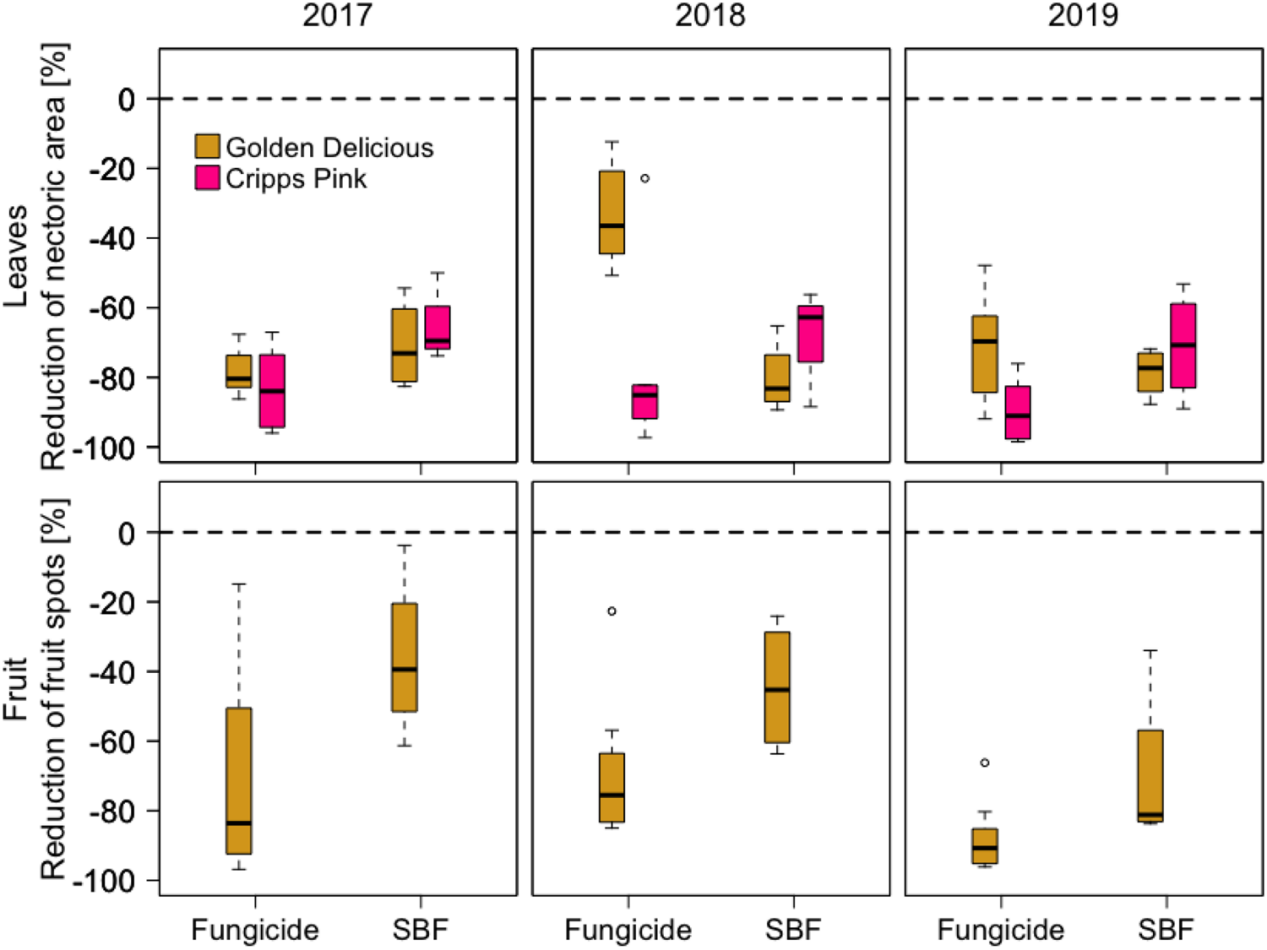
Relative reduction of Alternaria-associated leaf blotch and fruit spots using fungicides or sulphur-based leaf fertilizers (SBF). Shown are fungicide and SBF effectiveness in the two apple varieties ‘Golden Delicious’ (orange; fertilizer: Sulfate-Mix) and ‘Cripps Pink’ (Pink Lady^®^’; pink; fertilizer: microfine sulphur) compared to conventional practise (control, dotted line at zero). The field trial was performed in a randomized block design (n = 4 per treatment) from 2017 to 2019 at Laimburg Research Center, South Tyrol, Italy. Leaf blotch reduction in both varieties was significant across all years (*p* < 0.0001, Supplementary Table 2), while the reduction in fruit spots was only significant for ‘Golden Delicious’ (Supplementary Table 3). Fruit spots on ‘Cripps Pink’ were very rare to absent (control: mean number of spots fruit^-1^ ± SE: 0.0092 ± 0.0049; see Supplementary Fig. 14), which made it inappropriate to calculate relative reductions.

To validate our hypothesis, we conducted a three-year field experiment in apple orchards. On mature trees of both ‘Golden Delicious’ and ‘Cripps Pink’ varieties, we applied either custom-made sulphur-based leaf fertilizers without fungicidal effect (tested with in vitro fungicidal activity screening, Supplementary Table 1, Supplementary Fig. 15), commonly used fungicides, or neither (control), under conventional (‘integrated production’) cultivation practice. Over three years, the leaf fertilizers reduced the Alternaria leaf blotch by on average 72.7% (SE: ± 2.5) for both apple varieties (Fig. 3, *p* < 0.001; Supplementary Table 2). This reduction was the same as achieved by commonly used fungicides (72.7%, SE: ± 3.3). The sulphur-based leaf fertilizers also reduced fruit spots on ‘Golden Delicious’ (50.2%, SE: ± 7.3, *p* < 0.001, Supplementary Table 3), while the fungicides led to a 75.9% (SE: ± 4.6) reduction on average (Fig. 3, *p* < 0.001; Supplementary Table 2). In contrast, fruit spots in ‘Cripps Pink’ were rare (111-fold lower occurrence than for ‘Golden Delicious’) and a reductive effect of leaf-fertilizers on fruit spots could not be detected (*p* = 0.721). For ‘Golden Delicious’ we found a positive correlation between leaf blotch and the fruit spots in all three years (r = 0.53 to 0.78, Supplementary Fig. 3; Supplementary Table 4).

## Discussion

Based on our study, we have found evidence that challenges the widely held notion that Alternaria spp. is the primary and direct cause of Alternaria leaf blotch on apple and suggests that the disease is more complex than previously thought. Our findings contradict the idea that the fungus acts as a primary pathogen, as artificial inoculation of healthy tissue failed and fruit spots did not expand during storage. Additionally, we observed a strong association between low leaf nutrient concentrations and disease incidence.

Moreover, our study showed that simple sulphur-based leaf fertilizers, that do not inhibit fungal growth, can reduce Alternaria leaf blotch with the same efficacy as fungicides. Since the fertilizers strengthen the physiological condition of the plant, and do not act directly on the fungus, we suggest that it treats the primary cause of the disease, and that Alternaria infection is a secondary symptom. Based on this, we propose the new synthesis that physiological leaf blotch and Alternaria leaf blotch are not distinct diseases but rather part of a continuum, we call the "Alternaria-complex". This continuum conception recognizes the multifactorial nature of the disease induction and proliferation, and highlights the importance of considering both the physiological and fungal factors involved in disease development (Fig. 1).

We argue that the ubiquitous Alternaria spores colonize any pre-existing leaf necrosis and cannot initiate these necroses on their own. Instead, the initial leaf spots are primarily caused by leaf nutrient imbalances (Fig. 1c-i). However, initial leaf necroses can also be formed through other mechanisms, e.g. particular active agents sprayed during particular weather conditions can have phytotoxic effects (Fig. 1c-ii)^37–39^. Independently of the cause of the initial leaf spots, the ubiquitous Alternaria spores will subsequently colonize and enlarge the existing necroses (Fig. 1c-iii), potentially through the release of toxins (e.g. alternariol, altenuene, tenuazonic acid), leading to premature defoliation and yield loss ^40–44^. The same mechanism likely applies for fruit spot formation and only pre-damaged lenticels can be colonized by *Alternaria spp.* (Fig. 1d). This opportunistic pathogenic role of *Alternaria spp.* in disease progression explains why different fungicides also reduce the symptoms on leaves and fruits, but a primary Alternaria infection is not the initial cause.

This synthesis represents a significant improvement over the original competing hypotheses, as it provides a comprehensive explanation for the inconsistent and complex results related to the Alternaria disease (see Supplementary "Discussion: Two inadequate hypotheses").

We want to acknowledge that our infection experiments were conducted under controlled conditions and with a limited number of isolates, and thus represent only a fraction of possible interactions between *Alternaria spp*. and the apple host plant. Moreover, significant variability exists in the results of other infection experiments with *Alternaria spp.,* where some studies have reported failed artificial inoculation with no symptoms or inconsistencies^14,45–47^, while others have reported successful inoculation with symptoms^15,48–50^. The variable pathogenicity (successful vs. failed infection) of *Alternaria spp.* is typically explained by “pathotypes” of Alternaria strains, specifically *A. alternata*, which can produce different metabolites such as host-specific toxins that act as virulence factors or colonization factors like tenuazonic acid and alteraniol^41,42,51,52^. These toxins can trigger cell death and necrosis within hours when applied directly to plant tissue^40,44,53^. Unfortunately, our artificial inoculation study was conducted without metabolic characterization of the isolates used, and the failure of inoculation may be due to a lower virulence of these isolates^54^.

Given the current state of Alternaria research, we believe it is important to reexamine the emphasis placed on toxins and toxicity tests, particularly in regards to the characterization of virulence in *Alternaria spp.* and for the fulfillment of Koch's postulates. We believe that a critical examination of this method is necessary to ensure accurate interpretation of the infection biology of *Alternaria spp.* In “detached-leaf bioassays” or “pathogenicity bioassays” this direct toxin effect is used to infer the pathogenicity (pathotype vs. non-pathotype) of Alternaria strains^9,55,56^. Nevertheless, these assays can pose some challenges, because they (a) do not trace the biological process of infection, i.e. germination of spores and invasion of hyphae into the tissue of the host to “establishing contact and procure nutrients”^1^. This is because pathogenicity assays are typically performed on wounded tissue, sometimes with the purified toxins and without the fungus^9,23,40,42,48,53,55,57^. Furthermore, (b) the leaf necroses provoked in these assays (veinal, extensive necrosis) are symptomatically very different from leaf blotch in the field (concentric spots)^45,46,55,56,58^. Finally, (c) these necroses remain constant in size and do not enlarge when mycelial plugs were used as inoculum^47,48^. Overall, it's important to recognize that pathogenicity assays have limitations when it comes to determining whether *Alternaria spp.* can effectively infect host plants. Additionally, it's worth noting that there's no definitive evidence of primary infection (i.e., the penetration of hyphae into healthy tissue) by *Alternaria spp.* on apple plants. The differences between the “toxin effect”, “infection”, “secondary pathogen” and “primary pathogen” may seem subtle, but to avoid ineffective treatment, it is fundamental to understand the infection biology of a pathogen. For *Alternaria*, current practice has led to a misclassification of an opportunistic secondary pathogen as a primary pathogen, leading to unnecessary and unspecific treatment with fungicides.

Importantly, a strong analogy exists between “Alternaria-disease” on apple and “Alternaria-disease” on other crops. On potato, spores of *Alternaria solani*, thought to cause early blight, are produced mainly on dead or dying leaves^7^. Moreover, early blight is also associated with heat stress and nutrient deficiency^59,60^. In several crops, fungicide treatments have only poor efficacy, while magnesium (potato, poppy) and zinc (tomato) applications did control early blight indicating that a similar physiological mechanism may operate here as in apple. Likewise, foliar application of MgSO_4_ on poppy reduced disease severity of different mold fungi, including *Alternaria brassicae f. somniferi*^61^*.* For cotton it has been shown that chilling stress is the key predisposing factor for an infection with *A. alternata*^3^*.* In summary, several studies have demonstrated that Alternaria is related to nutrient deficiency or that infestation can be controlled with fertilizers^62–67^, but also other stresses are related to *Alternaria spp.*, such as drought, senescence, virus and insect damages or chilling stress ^3,7,68–71^. Hence, several abiotic and biotic (e.g. mites in our results) plant stresses can trigger colonization by *Alternaria spp.* also in other plants.

The association of *Alternaria spp.* to different type of stress is usually explained by a higher susceptibility due to a “weakened” host^2,7^. However, the term “weakened” addresses various changes in physiological processes, such as stress response mechanisms ^72–74^. The ‘weakened’ host hypothesis, also implies that an infection or penetration of fresh plant tissue can then take place, which is not in line with the saprophytic lifestyle of *Alternaria spp.*^1,2,7^. By contrast, we hypothesize that dead plant tissue is the key precondition for the colonization of *Alternaria spp.* on apple and likely also for other plants. The new hypothesis explains why a wide range of different stresses is associated with *Alternaria spp*.: all these stresses lead to necrosis which can be subsequently colonized by *Alternaria spp*., a primarily saprophytic fungus.

We are aware of the multifactorial interactions among different *Alternaria* species, different host plants with various cultivars. Although we did not test all susceptible varieties, we believe that our results are representative, as the majority of these varieties are genetically related, and the disease dynamics are likely to be similar. On the other hand, a large body of research is focusing on the “pathogenicity” of different *Alternaria* species/isolates (pathotypes) by focusing on the toxins produced or the related gene loci^6,44,75–78^. There are numerous *Alternaria* phytotoxins that have been described, and recently a new putative toxin was discovered to be produced in 14 different isolates^44,79^. The new toxin was not related to known Alternaria-toxin gene regions (AMT1, AMT4, AMT14), underlying the complexity of research on *Alternaria* pathogenicity. However, we argue that the pathotype typology and their potential to produce toxins cannot explain why fungicides and fertilizer-treatments equally reduced the symptoms. It would imply, that all the different types of fertilizer and nutrients (see references above; N, P, K, Mg, Zn, S, Mn) can “inactivate” multiple and highly effective toxins (necrosis within few hours) whose cell-damaging effect is believed to be a the key process for infection ^40,41,80,81^ (see also Supplementary "Discussion").

Although the ultimate causes of the leaf nutrient imbalances needs further investigation, climatic factors and particularly heat waves and subsequent drops in temperature have been implicated as key drivers^17,31^. Rising temperatures and an increasing frequency of heat periods and weather extremes in the study region are consistent with the increased frequency and spatial distribution (confined to hot valley bottoms) of Alternaria leaf blotch and the first appearance of fruit spots 22 years ago (in 1999)^82^. Moreover, the continuing expansion of leaf blotch to regions of (less warm) temperate climates points to climate change as a key driver ^23^. However, environmental and physiological mechanisms responsible for low Mn, S and Mg availability, uptake, leaching or sequestration remain poorly understood. Variable soil water status (drought vs. waterlogged) and unbalanced irrigation regimes can be potential drivers as they strongly influence the availability of nutrients, in particular manganese ^35^. Importantly, if the Alternaria-complex is indeed driven by climate change induced weather extremes, its importance for apple production will probably increase substantially in the near future.

In summary, our results show that *Alternaria spp.* is not the primary cause for Alternaria leaf blotch and fruit spots but an opportunistic pathogen. *Alternaria spp.* only colonizes pre-existing necrotic tissue of apple leaves and fruits. These pre-existing necroses are caused by a physiological nutrient disorder (involving S, Mn and Mg), first appearing as physiological leaf blotch. However, also other causes (e.g. red mites, phytotoxicity of active agents) can trigger initial necroses and thus act as a precondition for the colonization by *Alternaria spp.* Finally, we demonstrated that sulphur-based leaf fertilizers can be used as a simple preventive method to control Alternaria-associated diseases. The application of these leaf fertilizers can strongly reduce global fungicide use in apple production The potential impact will be strongly exacerbated if the same mechanisms also applies in other crops, as we hypothesize, and can likewise be treated by leaf-fertilizer treatment.

## Methods

### Field trials and Alternaria leaf blotch evaluation

All field trials were performed from 2017 to 2019 in the apple orchards of Laimburg Research Center, South Tyrol, Italy (224 m.a.s.l; 46.3825°/11.2887°). We established our experiments in two blocks, the ‘Golden Delicious’ block (planted 2012) and the ‘Cripps Pink’ (‘Pink Lady^®^’) block (planted 2000). Rows of mature apple trees were oriented from north to south and grown as slender spindle of 3,5 m height. We set up the experiments as randomized block design with 4 replicates, where a row constitutes a block. To minimize the influence of neighbouring treatments, we included two “buffer-rows” between the blocks. The plots within a block consisted of 15 consecutive trees. The first three and the last three trees of a plot, bordering the other plots, were not considered for evaluation.

For the evaluation of the treatments (see below), fruits and leaves were collected during the common harvest periods (Golden Delicious: 19th Sept. 2017, 19th Sept. 2018, 26th Sept. 2019; ‘Cripps Pink’: 7th Nov. 2017, 8th Nov. 2018, 15th Nov. 2019). From each plot we collected randomly two boxes of apples (~ 50–65 apples per box), one for each tree side (west and east). Fruits were immediately transferred to the cold storage cell (2.5 °C and 90% RH) until further evaluation. Leaves were collected randomly on the same day from the same trees, 100 leaves per plot (replicate) divided equally over the tree sides (50 leaves per side) and put in paper bags. To quantify “Alternaria” leaf blotch each leaf was analysed visually in the lab and assigned to an infestation class, corresponding to the percentual necrotic area of a leaf (0%, 0–5%, 5–10%, 10–15%, 15–20%, 20–30%, > 30%). Fruits were also assigned to infestation classes, considering the number of detected fruit spots per apple (0, 1–2, 3–5, > 5). Results were pooled for each plot, representing one replicate/data point. Fruit spot was almost absent in the fungicides treatment of ‘Cripps Pink’ and was therefore not evaluated.

#### Experimental treatments

Orchards were managed according to the guidelines for the integrated cultivation of pome fruits with the exception that no fertilizers and no specific Alternaria-active agents were applied^83^. Thus, our control plots represent integrated management without specific Alternaria treatments, while the other experimental plots were: integrated management plus corresponding experimental treatment. The tested treatments were developed based on previous results and field observation (see Supplementary Fig. 13). Our treatment against Alternaria leaf blotch was for Golden Delicious the sulphate-mix (SBF, see Supplementary Table 1 for composition). For ‘Cripps Pink’ we used in 2017 the sulphur suspension Thiopron^®^ (UPL Italia s.r.l; 2017: 0.605 l/hl = 500 g S/hl). As Thiopron^®^ caused a white coating on apples, in conflict with the selling criteria, we reduced the applied amount in 2018 (0.242 l/hl = 200 g S/hl) and substituted in 2019 Thiopron^®^ by another common Sulphur product (microfine sulphur), Thiovit^®^ (Syngenta Italia s.p.a). Thiovit^®^ was applied according to the recommended dosage (240 g/hl = 190 g S/hl). This shift to another sulphur product had no fundamental influence on our results as the effect on leaf blotch is independent of the sulphur type/product (see Supplementary Fig. 13). All previous described treatments we will call “sulfur-based leaf fertilizer” (SBF). Application frequency per season was for Golden Delicious 4 applications in 2017 and 5 applications in 2018 and 2019. For ‘Cripps Pink’, the application frequency was 6 times per season in all three years (2017–2019; see Supplementary Table 1). All treatments were applied by an experimental orchard sprayer (axial fan, injector nozzles) with a used water amount of 15 hl/ha.

To compare the effectiveness of the SBF to current practice, we also monitored treatments where several fungicides were applied to control Alternaria leaf blotch and fruit spot. We tested two active agents with two different modes of action on ‘Golden Delicious’: (I) Potassium phosphonate (FRAC code: P 07; 2017: “FosfiD'oR 250”, Orius; 2018–2019: “Century Pro”, BASF) and (II) Difenoconazol (FRAC code: 3; 2017–2019: “Score 10 WG”, Syngenta). Applied dosages were: FosfiD'oR 250: 3 L/ha (45.3% Potassium phosphonate), Century Pro: 1.9 L/ha (50.4% Potassium phosphonate), Score 10 WG.: 750 g/ha (10% Difenoconazol). Each of these fungicides was tested separately. As ‘Cripps Pink’ has a much longer growing period (harvest in November) and frequency of application is limited by legal regulations for each active agent to a certain number, we tested a Fungicide-Mix treatment. The Fungicide-Mix included the following fungicides: Syllit^®^ 65 (UPL), with 1,38 kg/ha (65% Dodine), Polyram^®^ DF (BASF), with 2,6 kg/ha (70% Metiram), Geoxe^®^ (Syngenta), with 0,45 kg/ha (50% Fludioxonil). All fungicides were applied according to good agricultural practice, usually after rainfalls when the risk of fungal infections is high. Treatment frequency of fungicides per growing season was for ‘Golden Delicious’ 2017: 8, 2018: 5, 2019: 11, and for ‘Cripps Pink’: 2017: 11, 2018: 7, 2019: 8. For statistics, results of all fungicide plots were included and thus represent an average effect of different fungicides (‘Golden Delicious’: 2 active agents; ‘Cripps Pink’: 4 active agents).

#### Experiment on sulphur fertilizers

Within our field trial (experimental set-up as described above), we tested in 2017 additionally the effect of different leaf fertilizers and fertilizer concentrations on leaf blotch. Used fertilizers were: Thiopron^®^ (UPL Italia s.r.l), Fytofert^®^ S (Desangosse Italia s.r.l), Sulfate-mix (see Table S1). All three fertilizers were applied in two different concentrations, which were always corrected to receive a final concentration of 50 g sulphur hl^−1^ and 500 g sulphur hl^−1^ (Thiopron^®^: 61 ml hl^−1^ and 606 ml hl^−1^, 68 ml hl^−1^ and 685 ml hl^−1^, Sulfate-Mix: 500 g S hl^−1^ as described in Table S1, for 50 g S hl^−1^ we reduced MgSO_4_ to 318.8 g hl^-1^.

#### In vitro fungicidal activity screening

To test if the sulphate-mix (S-Mix, see Supplementary Table 1) has a fungicidal effect on *Alternaria spp*., we performed a fungicidal activity screening in the laboratory. For that, we used 6 × 4 microtiter plates in which we filled PDA as a control, PDA mixed with S-Mix (PDA + S-Mix) in its field-applied concentration (see above) and PDA mixed S-Mix with 1/10 of it used concentration (PDA + 1/10 S-Mix). All three media (PDA, PDA + S-Mix, PDA + 1/10 S-Mix) were inoculated with four different Alternaria isolates (A02/F172, A02/F152, A15/271, A15/31) with three replicates each (9 wells per isolate). After inoculation, plates were transferred in an incubator at 25 °C. Mycelia growth (diameter, mm) was measured after 24 h with a ruler and compared to the control (PDA). From each replicate diameter was determined with two measurements in an orthogonal arrangement (technical replicates). Mycelia growth was not reduced by 1/10 S-Mix and S-Mix in the media (PDA), but slightly increased (*p* < 0.001; Supplementary Fig. 13). Interactions between the media (concentration) and isolates were significant (*p* = 0.007) as growth of one isolate (A02/F172) did not show differences between the concentrations. Thus, S-Mix has no fungicidal effect.

### Leaf nutrient analysis

For nutrient analysis we sampled leaves form different plots/treatments within our field trial (2017 to 2019). Over the three years we sampled about 9900 leaves from 891 trees taken from 99 plots for ‘Golden Delicious’, for ‘Cripps Pink’ this was respectively 9100, 819 and 91. We pooled all leaves collected from one plot, thus each plot represents a replicate and a data point in the subsequent statistical analysis. All leaves were evaluated for leaf blotch as described above and subsequently analysed for the nutrient concentration. For that, leaves (100 per plot) were dried in an oven at 60 °C for at least 72 h in a compartment drier and petioles were removed before analysis. Chemical analysis was performed by the Laboratory for Soil and Plant analysis Laimburg (accredited according to ISO 17025:2005). Nitrogen was analysed with an elemental analyser according to DIN EN ISO 16634-1:2009 (according to Dumas). Other nutrients (P, K, Ca, Mg, B, Fe, Mn, Cu, Zn, S) were analysed with inductively coupled plasma optical emission spectrometry (ICP-OES) according EPA 3052 1996 + EPA 6010D 201. All given nutrient concentrations are per “dry weight”.

### Monitoring low versus high prevalence orchards

Apple orchards can differ dramatically in Alternaria leaf blotch and fruits spot infestation prevalence, even if orchards are in close vicinity and of same apple variety. To validate our leaf nutrient analysis, we selected four ‘Golden Delicious’ orchard pairs. So as to minimize the influence of management, the orchard pairs were selected based on two criteria: (I) Each pair was managed by the same farmer (three farmers) and (II) each orchard pair consisted of one orchard showing the last ~ 5 years no or only minimal infestation and an orchard characterized by regularly very strong infestation (natural abundance). One pair did not fulfil criterion I) as we paired the Laimburg orchards with an external orchard. From every orchard, leaves were sampled three-times in 2018 (25th May, 20th July, 7th Sept.) and two-times in 2019 (17th May, 9th July), with two replicates per orchard and time point and 50 leaves from 15 trees per replicate. On the first sampling date (25th May 2018) we took three replicates per orchard. Leaves were analysed for nutrient concentration, as described above.

### Alternaria infection biology trials

#### Spore suspension for inoculation

All the isolates used in the study were obtained from the fungal culture collection at Laimburg and were initially isolated from apple leaf blotch and fruit spots in various orchards located in South Tyrol, Italy. The phylogenetic classification of the isolates was determined using the method proposed by Armitage et al. in 2015, and the results revealed that two isolates belonged to *A. alternata sspp. tenuissima*, while the remaining two isolates belonged to *A. alternate sspp. arborescens*.

PDA plates (two per isolate) were inoculated with Alternaria isolates from the Laimburg collection and kept in an incubator at 25 °C. After one week, we cut a 1.5 cm^3^ cube from the grown mycelium and grinded it in a mortar with 200 µl of sterile 0.03% tween solution. After the mycelium was completely ground, we added 4.8 ml tween solution to the mortar. 750 µl of the mycelium suspension was pipetted to V8 plates (4 plates per isolate), which were previously covered with soaked (sterile water) cellophane discs. Closed plates (parafilm) were kept in a climate chamber at 25 °C and a light program of 8 h blacklight (Philipps TL-D 36W BLB 1SL/25) and 16 h darkness. After 3 weeks, the cellophane-mycelium layer was removed with a pair of tweezers and put together with 8 ml of the tween solution in a 50 ml falcon tube (two plates per tube). Falcons tubes were vortexed for one minute, 5 min set in a ultrasonic bath (40 kHz) and again vortexed for 1 min. To separate mycelium from spores, the falcon tube content was subsequently filtered (80 µm mesh) and the resultant liquid part analysed via haemocytometer (Fuchs-Rosenthal counting chamber) for spore density. All spore suspensions were diluted to a final density of 1.25 × 10^6^ spores/ml.

#### Leaf disc assay

With this experiment we wanted to test the influence of different leaf injuries on the infection with *Alternaria spp.* There were four different types of leaf damage treatments: “none” = healthy tissue, “1 h before” = injured with a pair of combination pliers one hour before inoculation, “7 days before” = injured with a pair of combination pliers 7 days before inoculation, “Mites” = leaves collected on the day of inoculation from an orchard currently attacked severely by European red mites (*Panonychus ulmi*). These collected leaves had a visible mite damage. All leaves were collected form the orchard of Laimburg Research Center, except the leaves attacked by mites derived from a close by farm (~ 3 km distance).

For the injury type “7 days before”, ‘Golden Delicious’ leaves were marked and injured on the trees with a pair of pliers, 7 days before the experiment started. The injured leaves, the healthy leaves and leaves damaged by mites were collected in the morning of the experimental inoculation (29th May 2019) and used to cut leaf discs with a cork borer (15 mm diameter). Four discs per leaf were cut randomly, avoiding the inclusion of the midrib. To reduce the potential presence of other pathogens and moulds, discs were dipped in 3% sodium hypochlorite solution and into 70% ethanol solution, each for two seconds and washed for 10 s in sterile water. Leaf discs belonging to the injury type “1 h before” were subsequently injured with a pair of pliers. We selected four different isolates, which belonged to the clades *A. alternata sspp. tenuissima* (A18/F01/14, A18/F03/09) and two *A. alternata sspp. arborescens* (A18/F05/11, A18/F10/14; according to Armitage et al.^75^) Isolate were originally isolated from different infested orchards of South Tyrol. Our experimental setup was a fully crossed design of the factors “Type of injury” and factor “isolates”, both with four different isolates, resulting in 16 treatment combinations. We used four replicates for each treatment combination (four petri plates), consisting of four technical replicates (four discs per plate).

Small petri plates (55 mm diameter) were filled with a layer of autoclaved glass beads (3 mm diameter) and sterile water (3 ml) until the beads were covered. In each plate we set 4 leaf discs, corresponding to the same treatment. According to the four isolates we had four different spore suspensions of which we applied 60 µl with a pipette on the injury of every leaf disc (centre). As a control (n = 4), we used sterile water which we applied as above on leaf discs of every “Type of injury”. In conclusion, each petri dish included leaf discs of one “Type of injury” and one isolate or pure water (control; in total 5 treatments × 4 isolates × 4 replicates = 80 dishes). Petri dishes were subsequently placed in a climate chamber (Proclimatic ACLIL 900 Lt) at 25 °C, 65% RH and a light program according to the concurrent day length (15.5 h light/8.5 h dark, 5000 lx). Due to evaporative loss, we added on day 3, 6 and 9, 2 ml sterile water to every plate.

After 13 days (12th June 2019), every disc was evaluated for necrotic leaf blotch according to the following classes [% necrotic area]:0, 0–1, 1–5, 5–10-10–15, 15–20, 20–30, > 30. The transformation into numeric data was done as described above (main field experiment). Note: we evaluated the change in necrotic area relative to the necrotic area of the corresponding controls, which differed already before the inoculation due to the different “Type of injury” treatments.

#### Inoculation of apples

Mature ‘Golden Delicious’ apples were harvested at Laimburg Research Centre on 18th Sept 2019. In the lab, apple surface was cleaned with paper towels drenched in 70% ethanol solution. With a marker pen, the apple surface was divided in three equal spherical segments. Each segment‚ was assigned to one of the three injury types (treatment, n = 4): Needle = injured with syringe needle (5 punctures ~ 5 mm deep), Sand paper = injured with a sand paper (~ 6 cm^2^ of injured apple surface), None = untreated healthy tissue. By means of a brush, the injured areas and the healthy tissue were inoculated with a spore suspension (isolate A18/F10/14, *A. alternata sspp. arborescens*). As a control, we used sterile water which we applied analogously as the spore suspension on four additional apples with the same injury types. Apples were transferred to a plastic box with water-soaked paper towels on the bottom to increase air humidity. The apples were placed on a metal grid in the box to keep the apples dry. Boxes were coverd with a lid (not airtight) set in a climate chamber (Proclimatic ACLIL 900 Lt, 25 °C, 65% RH) and a light program according to the concurrent day length (12 h light/12 h dark). Water was refilled on day 4 and 8 to keep the towels wet. After 12 days, apples were controlled for fruits spots and necrosis formation as before.

#### Fruit storage experiment

We wanted to test if (a) fruit spots increase in number and size during storage and (b) if healthy apples can be infected by nearby infected apples during storage. For that, ‘Golden Delicious’ apples were harvested in September (common harvest date) from an orchard with severe Alternaria fruits spot infestation (mean: 10 spots per apple). Healthy apples were taken from another orchard with no infestation. Healthy apples with no fruit spots were placed together with the infected apples in plastic boxes (27th Sept. 2000). Both boxes were stored at room temperature (15–20 °C), one at 65% RH (10 infested, 15 healthy apples) and the other at 100% RH (11 infested, 10 healthy apples), respectively. After two months (25th Nov. 2000), apples were controlled again for fruit spots and compared to those before the storage.

### Alternaria colonization trials

#### Artificial leaf blotch field trial

To test if *Alternaria spp.* colonizes necrotic tissue, we provoked necrotic leaf spots artificially in the field. For that, the herbicide Spotlight^®^ Plus (Belchim; Carfentrazone-ethyl; concentration: 3 ml/L) was sprayed by means of a hand sprayer on ‘Golden Delicious’ leaves in the Laimburg orchard. Six trees per plot (n = 4) were treated on 8th July 2019 within our main field trial. The resulting artificial leaf spots were clearly distinguishable from natural leaf blotch. On 3rd September and 14th October 2019, five leaves per plot with clear symptoms were collected from the herbicide treated plots and the control plots with natural leaf blotch. Collected fresh leaves were scanned with a copy machine to create colour images (300 dpi). Based on the colour images, we determined the size (cm^2^) of the necrotic spot, by means the ImageJ software^84^. After scanning, leaves were dried within paper bags at 60 °C for at least 72 h in a compartment drier. Next, spores were washed from the dried leaves as follows: dried leaves of a plot were crushed and filled together in a 200 ml beaker with 60 ml deionized water, each. Beakers were shaken for 2 min (200 rpm), subsequently transferred for three minutes in an ultrasonic bath (40 kHz) and again shaken for 5 min (200 rpm). The water-leaf mix was filtered (80 µm mesh) and the resulting filtrate was used to analyse the spore density by means of a haemocytometer (Fuchs-Rosenthal counting chamber).

### Statistical analysis

The following analyses were based on linear mixed-effects model (LMM) analyses. All analyses were conducted in R version 4.0.2(R Core Team, 2019). Random Forest analyses were run using the randomForest package version 4.6-14^85^. Linear mixed models were fit using nlme version 3.1-148, and principle component analysis was conducted using the vegan package version 2.5-6^86,87^.

#### Main field experiment

For graphical and statistical analysis, the ordinal leaf blotch and fruit spot data was transformed to continuous data (percentage), by using the class midpoints to calculate the weighted means (Mean necrotic area [%], Mean number of fruit spots per apple) for each tree side of a plot. The reduction in leaf blotch and fruit spots was calculated by: (Mean necrotic area treatment / Mean necrotic area control)-1)*100.

The effect of SBF and fungicides (treatments) on leaf blotch and fruit spot, were analysed separately for each of the two treatments and each apple variety (‘Golden Delicious’ and ‘Cripps Pink’), respectively. “Necrotic leaf area” (%) and “Number of fruit spots” (# fruit^-1^) were used as response variables, respectively. The fixed effect was “Treatment” (SBF vs. control or Fungicide vs. control). As we had data from multiple years we specified “Year” (2017–2019) and Block (Replicate; n = 4) as nested random effects with separate slopes (Year | Block).

#### Monitoring low versus high prevalence orchards

To test if the susceptibility to Alternaria leaf blotch is linked to nutrient concentration (Mg, Mn, S), we used LMMs with Mg, Mn, S concentration as the response variables. “Orchard type” (high vs. low prevalence) was set as fixed effect. “Date” (n = 5, with 2 replicates each per orchard) and „Orchard ID” (2018: n = 8; 2019: n = 6) were specified as random effect with separate slopes (Date|Orchard ID).

#### Spore density of artificial leaf blotch

The “spore density” of Alternaroid-spores extracted from artificially (herbicide) provoked leaf spots and natural leaf blotch was set as dependent variable. Fixed effects were “Leaf necrosis type” (artificial vs. natural) and “Date” of leaf collection (3. Sept. 2019 vs. 14. Oct. 2019) with their interaction. “Block” (Replicate; n = 4) was set as random effect.

#### Leaf disc assay (LDA)

We used the relative necrotic leaf area of leaf discs as depended variable. The values for each replicate represent the mean of the corresponding technical replicates (n = 4). “Injury type” (“none” vs. “one hour before” vs. “7 days before” vs. “Mites”) and “Isolate” (A18/F01/14 vs. A18/F03/09 vs. A18/F05/11 vs. A18/F10/14) were set as fixed effects, as well as their potential interaction. “Replicate” (n = 4) was specified as random effect.

#### Leaf nutrient analysis from main field experiment

To link leaf nutrient concentrations to leaf blotch prevalence we ran Random Forest analyses for the Golden and Pink varieties separately^88^. For each forest, we grew 10.000 trees, randomly sampling 4 predictors for splitting at each node. Variables were ranked by their mean decrease in node impurity, as measured by residual sums of squares, and those with a disproportionate impact were selected for further analysis after visual inspection.

Subsequently the selected nutrients were used in overall linear mixed models to test their effects on (log-transformed) leaf blotch severity in the two varieties to maximize statistical power. Random effects were included to account for repeated observations on the same plots across years. Fixed effects included the variety, the selected leaf nutrients and the two-way interactions between each leaf nutrient and variety. The latter allows each variety to have a different relationship between each nutrient and leaf blotch. There was too little data to explore further interactions among nutrients.

Two of the selected predictors (S and Mg) were strongly collinear (Supplementary Fig. 4) and including both in the same model led to unstable estimates of regression coefficients. To get around this issue we used two approaches. First, we ran a principal component analysis on just the S and Mg data. The first axis explained most of the variation (90.1%) in these two elements, as expected. Subsequently, the PCA axes (1 & 2) scores were used as predictors in the LMM (mod1, Supplementary Table 5) to capture simultaneously the effects of S and Mg on leaf blotch. Secondly, we fitted two LMMs excluding either Mg (mod2) or S (mod3) as predictors and compared the model fit based on AIC values^89^. We further simplified the best fit model (mod2), using likelihood-ratio tests (mod2a).

### Statement on the collection of plant material

The plant materials used in this study were sourced from controlled cultivation and all collection were made in accordance with institutional, national and international guidelines for the collection of wild plants.

## Supplementary Information

Supplementary Information.

## Data Availability

The datasets used and/or analysed during the current study is freely accessible at: 10.5281/zenodo.7963642.
