# Publisher Correction: Fungicide-free management of Alternaria leaf blotch and fruit spot on apple indicates *Alternaria spp.* as secondary colonizer

**DOI:** 10.1038/s41598-023-36760-7

**Published:** 2023-06-13

**Authors:** Ulrich E. Prechsl, Werner Rizzoli, Klaus Marschall, E. R. Jasper Wubs

**Affiliations:** 1 Research Centre Laimburg, Laimburg 6, 39040 Auer/Ora, BZ Italy; 2 Sustainable Agroecosystems Group, Institute of Agricultural Sciences, Department of Environmental Systems Science, ETH Zürich, Universitätstrasse 2, 8092 Zürich, Switzerland; 3 Department of Terrestrial Ecology, Netherlands Institute of Ecology (NIOO-KNAW), P.O. Box 50, 6700 AB Wageningen, The Netherlands; 4 Present Address: Terra Institute, Säbenertorgasse 2, 39042 Brixen, BZ Italy

Correction to: *Scientific Reports* 10.1038/s41598-023-35448-2, published online 24 May 2023

The original version of this Article contained errors. Werner Rizzoli and Klaus Marschall were incorrectly affiliated with ‘Terra Institute, Säbenertorgasse 2, 39042, Brixen, BZ, Italy’, which is a present affiliation for Ulrich E. Prechsl.

The correct affiliations for these authors are listed below.

**Ulrich E. Prechsl**

Research Centre Laimburg, Laimburg 6, 39040, Auer/Ora, BZ, Italy

Present address: Terra Institute, Säbenertorgasse 2, 39042, Brixen, BZ, Italy

**Werner Rizzoli**

Research Centre Laimburg, Laimburg 6, 39040, Auer/Ora, BZ, Italy

**Klaus Marschall**

Research Centre Laimburg, Laimburg 6, 39040, Auer/Ora, BZ, Italy

In addition, the email address of Ulrich E. Prechsl was incorrectly given as ‘Ulrich.Prechsl@laimburg.it’; the correct email address is ‘uli.prechsl@googlemail.com’.

The original Article has been corrected.

