# Supplementary material for: Fungicide-free management of Alternaria leaf blotch and fruit spot on apple indicates Alternaria spp. as secondary colonizer
Source: Sci Rep. 2023 May 24;13:8431. doi: 10.1038/s41598-023-35448-2 (PMC10209047; doi:10.1038/s41598-023-35448-2)
Supplement: Supplementary file 1 — Supplementary Information. [file 41598_2023_35448_MOESM1_ESM.docx]

Supplementary INFORMATION

of the article:

**Fungicide-free management of Alternaria leaf blotch and fruit spot on apple indicates *Alternaria spp.* as secondary colonizer**

DOI: 10.1038/s41598-023-35448-2

Ulrich E. Prechsl^1,*^, Werner Rizzoli^1^, Klaus Marschall^1^, E.R. Jasper Wubs^2,3^

^1^ Research Centre Laimburg, Laimburg 6, Auer/Ora, BZ 39040, Italy

^2^ Sustainable Agroecosystems Group, Institute of Agricultural Sciences, Department of

Environmental Systems Science, ETH Zurich, Universitatstrasse 2, 8092, Zurich, Switzerland

^3^ Netherlands Institute of Ecology (NIOO-KNAW), Department of Terrestrial Ecology, P.O.

Box 50, 6700 AB Wageningen, the Netherlands.

* Corresponding author: uli.prechsl@gmail.com

**Supplementary Results S1**

**Relationships between leaf nutrient content and leaf blotch severity**

The Random Forest analyses were conducted separately for `Golden Delicious´ and `Cripps Pink´ varieties highlighted S, Mg, Mn, P, and B as potentially strong predictors of leaf blotch severity (Supplementary Fig. 5). These predictors were jointly evaluated in overall linear mixed model analyses, including both varieties to maximise statistical power. As S and Mg were strongly collinear (r = 0.78) we fitted three linear mixed models. First, we ran a model where the effects of S and Mg were represented as PCA axes (Supplementary Table 5, mod1, Supplementary Fig. 6c), and then two further models where either S (mod2) or Mg (mod3) was included as a predictor. Based on AIC values both mod1 and mod2 are comparable (Supplementary Table 5), while mod3 has a substantially worse fit to the data (ΔAIC = 15-16). Both mod1 and mod2 lead to the same qualitative conclusions (Supplementary 5) and the estimated slopes were similar in magnitude (Supplementary Fig. 6a,b). The slope of the relationship between leaf blotch severity and S (Supplementary Fig. 6b, mod2) is much steeper than for Mg (Supplementary Fig. 6d, mod3). Together, these data suggest that sulphur, not magnesium, is the leaf nutrient reducing leaf blotch in both varieties most strongly (Fig. 3a).

In addition, low manganese concentrations were also associated with high leaf blotch severity, but only for Golden Delicious (Fig. 3b). Phosphorus levels were positively associated with leaf blotch severity (Supplementary Fig. 6f). However, the range of P concentrations in Golden was very small, and the model term p-value close to the threshold (p = 0.03), therefore we have less confidence in this result^1^. Boron concentrations did not significantly affect leaf blotch in the LMMs (Supplementary Table 5). From the above models, we prefer mod2, as it is the simplest and has the best fit to the data (Supplementary Table 5). If we further exclude the non-significant predictor B (mod2a, Supplementary Table 5), the effects of S and Mn become more pronounced (higher F-ratios), while the effect of P becomes non-significant. This further corroborates out interpretation that S and Mn are the key leaf elements associated with leaf blotch.

**Supplementary Results S2**

**High Alternaria leaf blotch prevalence orchards have leaf nutrient deficiencies.**

In 2018 and 2019 we analysed different `Golden Delicious´ orchards in the valley bottom of South Tyrol for nutrient concentrations in leaves. To reduce the influence of management, we selected “orchard-pairs”, with both orchards managed by the same farmer but contrasting strongly in Alternaria leaf blotch and fruit spot infestation (i.e. one high and one low prevalence orchard). From each orchard we collected leaves from 2 x 20 trees at different time points across the year (2018: 3 times; 2019: 2 times) with subsequent leaf nutrient analysis. High prevalence orchards which had Alternaria leaf blotch infestation every year showed significantly lower magnesium and manganese concentrations in the leaves (concentrations per dry weight, mean±SE Mn: 74.7±8.7 ppm; Mg: 0.37±0.019% w:w) than the low prevalence orchards (Mn: 131.9±9.9 ppm; Mg: 0.46±0.014%; Supplementary Fig. 7; Supplementary Table 6). Sulphur concentration was significantly lower in high prevalence orchards (S: 0.19±0.002%) than in low prevalence orchards (S: 0.21±0.006%). Other analysed nutrients (N, P, K, Ca, B, Fe, Cu, Zn) did not differ between high and low prevalence orchards (Supplementary Fig. 8, Supplementary Table 6). This analysis of different `Golden Delicious´ orchards confirms that sulphur, magnesium and especially manganese as putative drivers of leaf blotch formation (c.f. Supplementary Results S1).

**Supplementary Discussion**

Our new “Alternaria-complex” synthesis (Fig. 1) links two earlier hypotheses to explain and control Alternaria leaf blotch damage: Alternaria leaf blotch (hypothesis A) and the Physiological leaf blotch (hypothesis B). Both hypotheses on their own are insufficient to explain the process of disease development, as our data highlights.

*Two diseases, same symptoms*

For `Golden Delicious´, the most sensitive variety, first symptoms of “Alternaria” leaf blotch usually occur in late spring/early summer (June, July) with small brown leaf spots, which can increase in size rapidly (Supplementary Fig. 2). The typical blotches have a diameter of 2-3 cm with a dark brownish color, including several concentric rings (tree ring like) and a dark, black edging (Supplementary Fig. 1a). Over time, leaf spots can increase in number (>10 leaf^-1^) and turn into a greyish color (Supplementary Fig. 1b). Rainfall and heat periods in summer with subsequent drop of temperature increase the symptoms dramatically with severe defoliation as a consequence (Supplementary Fig. 1c)^2,3^. The description for symptoms and conditions favoring physiological leaf blotch are almost identical^4–6^. For `Cripps Pink´ (`Pink Lady®´), symptom appearance is lagged by about 4-6 weeks with a less rapid increase as in `Golden Delicious´ (Supplementary Fig 2). The first single fruit spots can appear in July, but a dramatic increase in number usually happens in the last two weeks before harvest (September; Supplementary Fig. 2).

*Two inadequate hypotheses*

Hypothesis A: Originally, it was thought Alternaria leaf blotch was due to a classical infection of *Alternaria spp.* (Fig. 1a), via cuticle, stomata and wounds^2,3,7,8^. Further it was thought that cell and membrane damaging toxins play a central role for the infection of healthy tissue^9–13^.

Hypothesis B: In contrast, the physiological leaf blotch of `Golden Delicious´ hypothesis (Fig. 1b), proposed that a physiological disorder underlies the formation of leaf blotch, which is triggered by heat periods in summer with subsequent rainfalls and drop in temperature^6^. However, also light intensity and soil moisture with subsequent hormonal disturbance (gibberellins) have been proposed as drivers^4,5,14^. The exact mechanism behind this disease was hitherto not understood well, but the involvement of magnesium and manganese was already proposed^4,5,14^.

Both hypotheses are not adequate since they are inconsistent with the data. Problems with hypothesis A are: We could not verify any indication of infection of healthy tissue, not on leaves nor on fruits. Even though *Alternaria spp.* grew after artificial inoculation with a very dense mycelium on top of the leaf surface (see Supplementary Fig. 1f), it did not penetrate and invade inside of the leaves. Invasion and growth of a pathogen within the plant tissue are key criteria for infection^15^. Our observation on missing infection is in line with the literature^16–20^. Within the genus of Alternaria, most strains are saprophytic and injuries and wounds to plants are described as prerequisite for pathogenic infection by *A. alternata and A. tenuissima*^3,15,21^. Hypothesis B on physiological leaf blotch, on the other hand, also has a fundamental inconsistency: fungicides (difenoconazole, potassium phosphonates), with different modes of action and free of Mn, Mg, Zn and S are also effective against leaf blotch. Thus, the cause of leaf blotch cannot be purely physiological.

*Synthesis: Alternaria complex*

Therefore, we suggest a new synthesis of physiological leaf blotch and Alternaria leaf blotch, which we call the “Alternaria-complex”. We hypothesize that both diseases are linked (Fig. 1c). First, environmental conditions, as discussed above, lead to depletion of leaf S, Mg and Mn content with physiological leaf blotch as a consequence (Fig. 1c-i). The resultant necrotic leaf spots are the prerequisite for the colonization of *Alternaria spp.* (**Fig. 1c-ii).** Once *Alternaria spp.* has established on the leaf spots, it has the potential to increase the necrotic area, likely by releasing cell wall and membrane damaging toxins^10,11,13^**.** Due to this strong linkage of the physiological component and the fungal component within the Alternaria-complex both fungicides and leaf-applied fertilizers can reduce leaf blotch. Even if both have completely different modes of action.

However, leaf necrosis can also be provoked by several other drivers*.* For instance, under certain conditions some active agents of agrochemicals (e.g. fungicides) can be toxic for plants (phytotoxicity) and provoke necrotic spots^22–24^. These spots can subsequently be colonized by *Alternaria spp.* **(Fig.** **1c-iii**) as we observed in our herbicide-trial **(Supplementary Fig. 12)**. The spore density of alternaroid spores (Alternaria-like) was 14 times higher on artificially provoked leaf necroses (herbicide, positive control) than on naturally occurring Alternaria leaf blotch. This clearly illustrates the opportunistic life strategy of *Alternaria spp.* as its presence is the consequence of leaf necrosis and not *vice versa*. However, what triggers this opportunistic organism to act as a secondary pathogen is still unclear. This is illustrated in our leaf disc assays. Alternaria isolates were only able to increase necrosis when leaves were pre-damaged by European red mites (*Panonychus ulmi*), but not when we created wounds (fresh and old) with a pair of pliers. The link between mites and Alternaria has also been observed under field conditions by other researchers^25^. In that field study, leaf blotch severity increased with increasing mite density, confirming the strong linkage between plant stresses and Alternaria leaf blotch. Our results suggest that the interaction with mites and herbicides can somehow trigger the change to a secondary pathogen, but mechanical damages do not. Generally, we think there are certain, as yet unknown, necrosis “qualities” that can trigger the switch to pathogenicity.

For effective management, the leaf-fertilizer treatments will probably have to be tailored to different crops and varieties*.* The two tested apple varieties showed important differences in the reaction to the leaf fertilizers. While `Cripps Pink´ can effectively be treated by simple sulphur leaf fertilizers, `Golden Delicious´ was only little affected by mere sulphur but showed a strong reduction in leaf blotch when treated with the Sulphate-Mix (containing S, Mg, and Mn, Supplementary Fig. 13). These observations are in line with our leaf nutrient analyses (Fig. 2, Supplementary Fig. 6, Supplementary Results S1). We found that low S-concentrations in leaf tissue were associated with leaf blotch in both varieties (Fig. 2a). In addition, low Mn-concentrations were only associated with leaf blotch in `Golden Delicious´, but not in `Cripps Pink´ (Fig 2b). This indicates that there are important differences in nutrient physiology and susceptibility to stresses on a variety-level. Interestingly, previous studies on `Golden Delicious´ did test Mg-, and Mn-treatments, but did not resolve if and what type of nutrient imbalance was responsible for the disease and did not develop an effective cure^5,8,14,26^. We explain the higher efficacy of our leaf-fertilizer treatment (Sulphate-Mix) on `Golden Delicious´ by a) a synergistic effect of the three combined sulphates and b) the higher nutrient concentration of the applied solution (Sulphate-Mix: 3,8% MgSO_4_ solution, see Supplementary Table 1) compared to earlier trials (1-2% MgSO_4_ solution).

In contrast to leaf blotch, the dynamics for fruit spot are less well understood. However, the consistent positive correlation between leaf blotch and fruits spot suggests a linkage between both symptoms. In addition, fruit spots always appear some weeks after the appearance of leaf blotch, but never *vice versa* (Supplementary Fig. 2). We believe that the underling mechanism behind this linkage is a strong inoculum formation (spores) on leaves of the surrounding tree with a maximum in August/September, analogous to the seasonal cycle of airborne spore density (pollen monitoring South Tyrol State Agency of Environmental Affairs). Released spores, primarily from leaf blotch but also from dead organic matter on the soil surface, can under certain pre-conditions colonize lenticels (periderm pores) of apple fruits and provoke fruit spots as a consequence (Fig. 1d). The likely preventative mechanism of our sulphur-based leaf fertilizers on fruit spots is thus via the reduction in leaf necrotic area, the substrate for *Alternaria spp.*, and thus inoculum density, while fungicides affect spore vitality (germination) directly. Both methods of control result in a lower prevalence of fruit spots. The latter also explains why the observed effect of the SBF was smaller on fruits than the effect of fungicides, because its effect on colonization is indirect.

We think that lenticel colonization with subsequent fruit spot formation results from the same principle as on leaves: *Alternaria spp.* colonizes only irritated or pre-damaged lenticels (Fig. 3d): these pre-conditions are necessary to allow Alternaria colonization. In our post-harvest experiment (Supplementary Fig. S11) fruit spots on infected apples did not increase (number nor size) during two-month storage. Moreover, healthy apples were not infected by close-by infected apples during two-month storage. If *Alternaria spp.* would be able to infect healthy tissue, it should expand during storage, as it is observed for other fungal diseases, such as bitter rot (*Neofabrea spp.*) or *Penicillium* rot (*Penicillium expansum*)^27^. In the field, fruit spot incidence can drastically increase within a few days (Supplementary Fig. 2). Analogous to leaf blotch, we have observed that this can be enhanced after certain management treatments (e.g. Captan, Chelat based leaf fertilizers (DTPA; EDTA), lime sulphur), often in combination with slight sunburn so that fruit spots appear more frequently on the sun-exposed side.

Thus, both on apple fruits and on leaves, pre-damages from different types of stresses seem to be obligate precursors for colonization of *Alternaria spp.*, a primarily saprotrophic fungus^3^*.*

**Supplementary References**

1. Zuur, A., Ieno, E. N., Walker, N., Saveliev, A. A. & Smith, G. M. *Mixed effects models and extensions in ecology with R*. (Springer Science & Business Media, 2009).

2. Filajdic, N. & Sutton, T. B. Influence of temperature and wetness duration on infection of apple leaves and virulence of different isolates of Alternaria mali. *Phytopathol. USA* **82**, 1279–1283 (1992).

3. Rotem, J. *The genus Alternaria: biology, epidemiology, and pathogenicity.* (American Phytopathological Society, 1994).

4. Back, C.-G., Lee, S.-Y., Kang, I.-K., Yoon, T.-M. & Jung, H.-Y. Occurrence and Analysis of Apple Blotch-like Symptoms on Apple Leaves. *Hortic. Sci. Technol.* **33**, 429–434 (2015).

5. Jonkers, H. Review on leaf spot and leaf drop: A physiological disorder of the ‘Golden Delicious’ apple. *Sci. Hortic.* **1**, 231–237 (1973).

6. Sutton, T. B. & Sanhueza, R. M. Necrotic leaf blotch of Golden delicious—Glomerella leaf spot: a resolution of common names. *Plant Dis.* **82**, 267–268 (1998).

7. Filajdić, N. & Sutton, T. B. Identification and distribution of Alternaria mali on apples in North Carolina and susceptibility of different varieties of apples to Alternaria blotch. *Plant Dis.* **75**, 1045–1048 (1991).

8. Sutton, T. B., Aldwinckle, H. S., Agnello, A. M. & Walgenbach, J. F. *Compendium of apple and pear diseases and pests*. (Am Phytopath Society, 2014).

9. Kodama, M. Evolution of pathogenicity in Alternaria plant pathogens. *J. Gen. Plant Pathol.* **85**, 471–474 (2019).

10. Kohmoto, K., Khan, I. D., Renbutsu, Y., Taniguchi, T. & Nishimura, S. Multiple host-specific toxins of Alternaria mali and their effect on the permeability of host cells. *Physiol. Plant Pathol.* **8**, 141–153 (1976).

11. Logrieco, A., Moretti, A. & Solfrizzo, M. Alternaria toxins and plant diseases: an overview of origin, occurrence and risks. *World Mycotoxin J.* **2**, 129–140 (2009).

12. Meena, M., Swapnil, P. & Upadhyay, R. S. Isolation, characterization and toxicological potential of Alternaria-mycotoxins (TeA, AOH and AME) in different Alternaria species from various regions of India. *Sci. Rep.* **7**, 1–19 (2017).

13. Meena, M. & Samal, S. Alternaria host-specific (HSTs) toxins: An overview of chemical characterization, target sites, regulation and their toxic effects. *Toxicol. Rep.* **6**, 745–758 (2019).

14. Jonkers, H. Leaf spot of the apple ’Golden Delicious’: A hormonal disorder. in *Symposium on Growth Regulators in Fruit Production 120* 253–253 (1981).

15. Agrios, George. *Plant Pathology - 5th Edition*. (Elsevier, 2005).

16. Dang, J. L. *et al.* Alternaria malicola sp. nov., a New Pathogen Causing Fruit Spot on Apple in China. *Plant Dis.* **102**, 1273–1282 (2018).

17. Dickens, J. S. W. & Cook, R. T. A. Japanese pear black spot and apple alternaria blotch. *EPPO Bull.* **25**, 651–659 (1995).

18. Gur, L., Reuveni, M. & Cohen, Y. Occurrence and etiology of Alternaria leaf blotch and fruit spot of apple caused by Alternaria alternata f. sp. mali on cv. Pink lady in Israel. *Eur. J. Plant Pathol.* **147**, 695–708 (2017).

19. Horlock, C. M. *Management of Alternaria leaf and fruit spot in apples*. (Horticulture Australia, 2006).

20. Toome-Heller, M., Baskarathevan, J., Burnip, G. & Alexander, B. First report of apple leaf blotch caused by Alternaria arborescens complex in New Zealand. *N. Z. J. Crop Hortic. Sci.* **46**, 354–359 (2018).

21. Thomma, B. P. H. J. Alternaria spp.: from general saprophyte to specific parasite. *Mol. Plant Pathol.* **4**, 225–236 (2003).

22. Dias, M. C. Phytotoxicity: An overview of the physiological responses of plants exposed to fungicides. *J. Bot.* **2012**, (2012).

23. Holb, I. J., Jong, P. F. D. & Heijne, B. Efficacy and phytotoxicity of lime sulphur in organic apple production. *Ann. Appl. Biol.* **142**, 225–233 (2003).

24. Simon, P. J. Necrotic leaf spot on apple leaf tissue: In vitro measurements. *Sci. Hortic.* **29**, 147–154 (1986).

25. Filajdic, N., Sutton, T. B., Walgenbach, J. F. & Unrath, C. R. The influence of European red mites on intensity of Alternaria blotch of apple and fruit quality and yield. *Plant Dis.* **79**, 683–690 (1995).

26. Mantinger, H., Vigl, J. & Demattio, S. Spritzversuch gegen vorzeitigen Blattfall bei Golden Delicious. *Obstbau/Weinbau* **15**, 105–106 (1978).

27. Zanella, A. *et al.* FRUDISTOR: The App for Determining Storage Disorders on Apples. http://www.frudistor.de/index.php?lang=EN (2021).


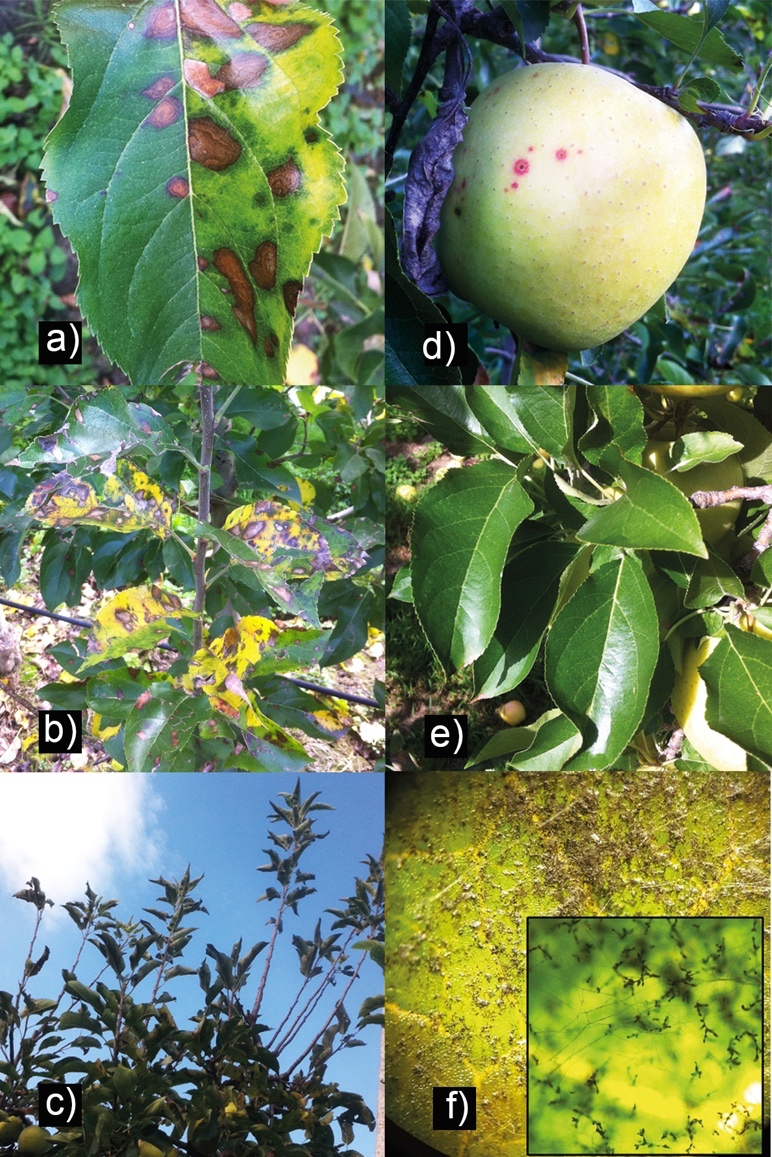


**Supplementary Figure 1:** Symptoms of Alternaria-associated disease on apple as observed in South Tyrol. a) Leaf blotch on `Golden Delicious´ at control management during the harvest period (20^th^ Sept. 2017). b) Advanced leaf blotch (20^th^ Sept. 2017) leads to yellowing and subsequent premature defoliation with up to 85% leaf drop. c) Premature leaf drop at water sprouts as a consequence of increasing leaf blotch. d) Fruit spot at `Golden Delicious´. e) `Golden Delicious´ leaves treated with sulphur-based fertilizers (SBF), during the harvest period (Sept. 2017). f) Dense Alternaria spp. mycelium with conidia chains after inoculation with spore suspension on the outside of the apple leaves (see leaf disc assay). There were no indications for an infection.


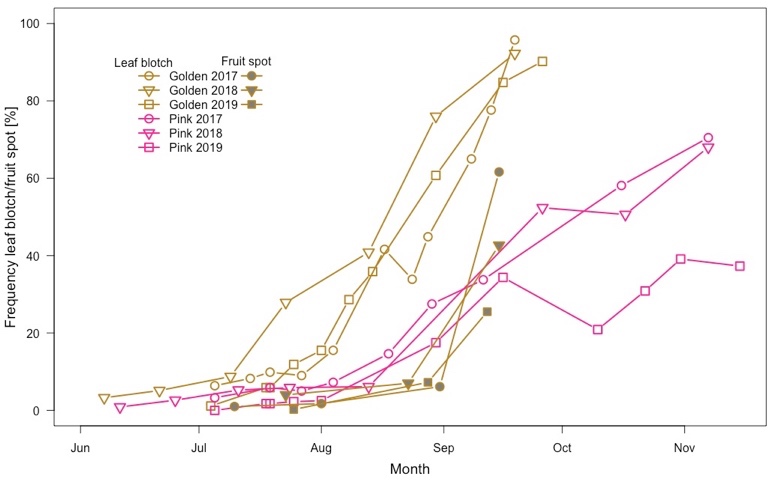


**Supplementary Figure 2:** Prevalence in Alternaria-associated leaf blotch and fruit spot. Frequency of leaves with symptoms (n=4 each based on 50 technical replicates) was evaluated for the apple varieties `Golden Delicious´ (light brown, open symbols) and `Cripps Pink´ (Pink Lady®´; pink, open symbols) at Laimburg Research Station, South Tyrol, Italy, for the years 2017-2019. Frequency of fruit spot was evaluated as well, but only for `Golden Delicious´ (light brown, closed symbols).


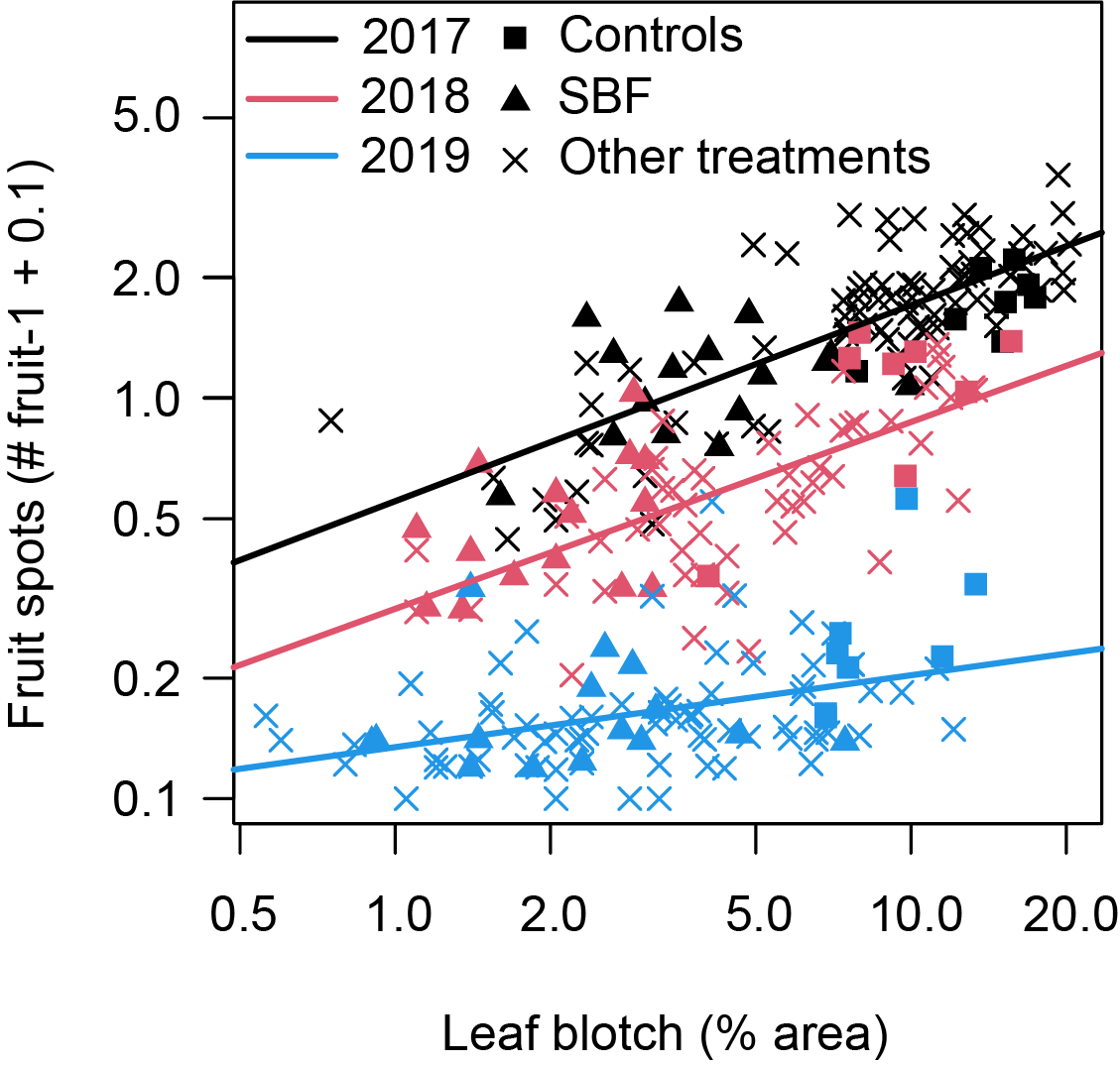


**Supplementary Figure 3:** Relationship between Alternaria fruit spots and Alternaria leaf blotch at the apple variety `Golden Delicious´. The analysis is based on symptoms during harvest in the years 2017-2019 (see **Supplementary Figure 1&14**). Symbols indicate different treatments. SBF = sulphur-based leaf fertilizer. See **Supplementary Table 4** for statistical analysis.


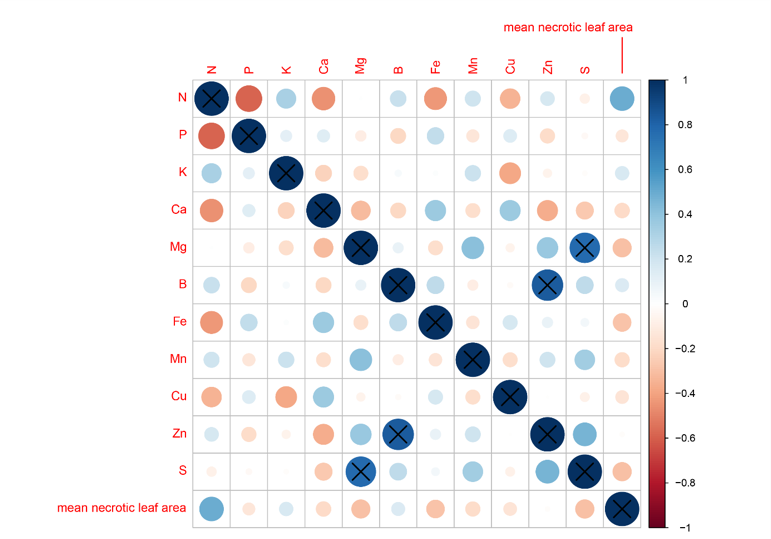


**Supplementary Figure 4:** Correlation plot for each of the leaf nutrients and leaf blotch severity (mean necrotic leaf area). Two pairs of predictors (S and Mg, B and Zn) have strong positive correlations (r > |0.7|, marked with black crosshairs).


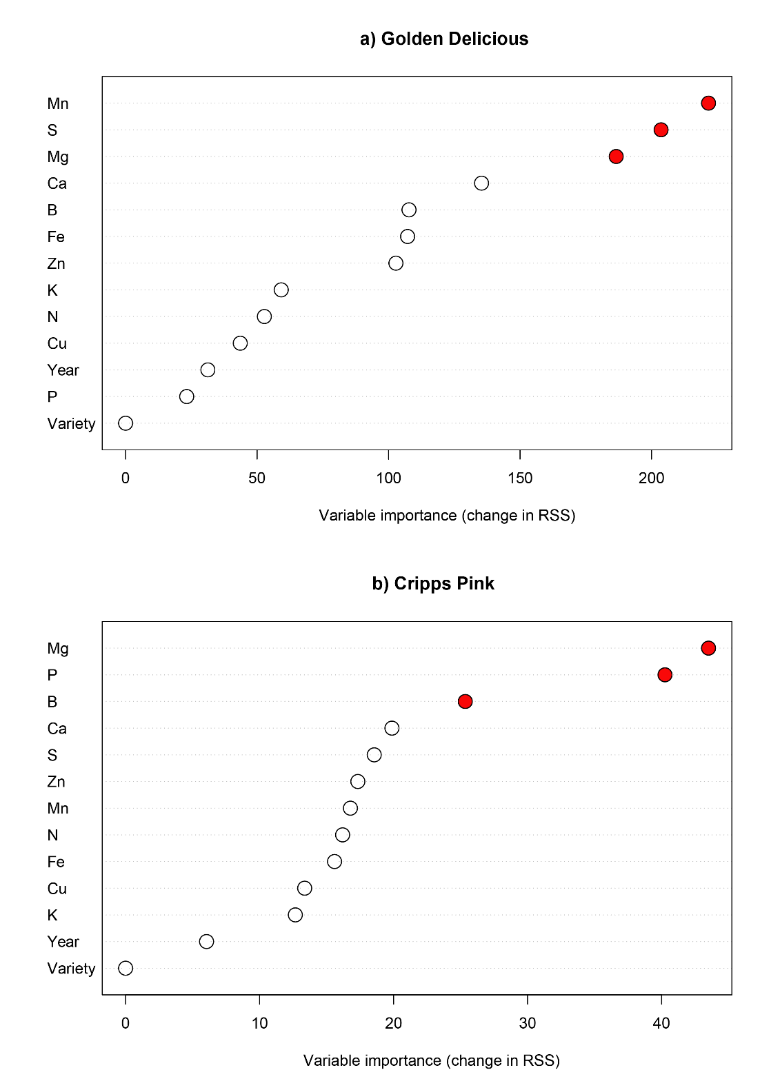


**Supplementary Figure 5:** Variable importance plots for the RandomForest analyses for `Golden Delicious´ (a) and `Cripps Pink´ (b) varieties separately. Predictors are ranked by their importance as measured by the mean decrease in residual sums of squares. Red circles indicate the predictors that were considered of disproportionate importance and selected for inclusion in downstream linear mixed model analysis.


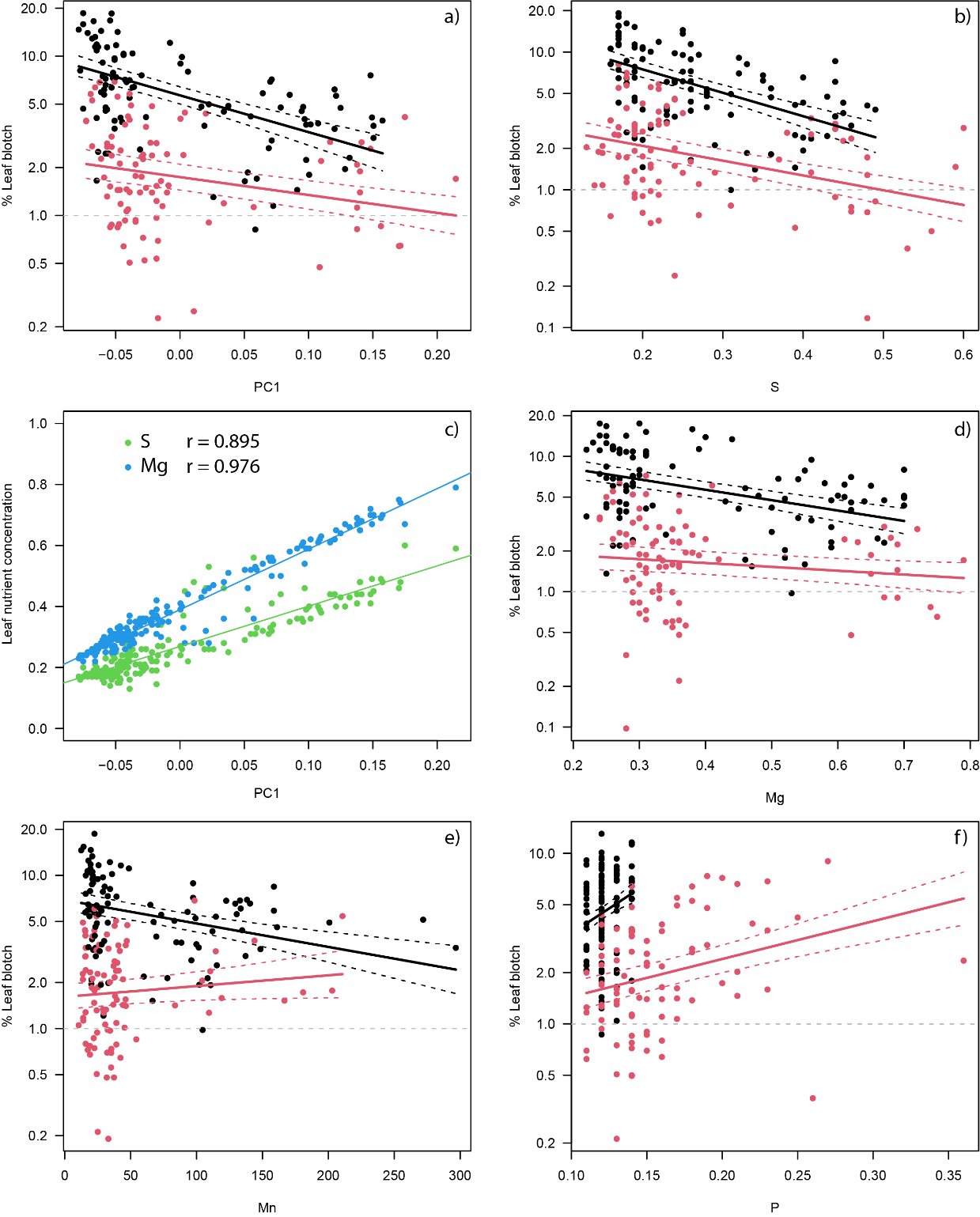


**Supplementary Figure 6:** Fitted relationships between blotch area and leaf nutrient concentration for `Golden Delicious´ (black) and `Cripps Pink´ (red) apple varieties. Note leaf blotch is plotted on a logarithmic scale. a) relationship with PCA axis 1 (model mod1), b) relation with S (mod2), c) relation between S and Mg with PCA axis 1 (mod1), d) relation with Mg (mod3), e) relation with Mn (mod2), f) relation with P (mod2). Panels b and e are the same as Fig. 1a, b respectively. For statistical analyses see **Supplementary Table 5** and the structure of the models (mod1, mod2, mod3).


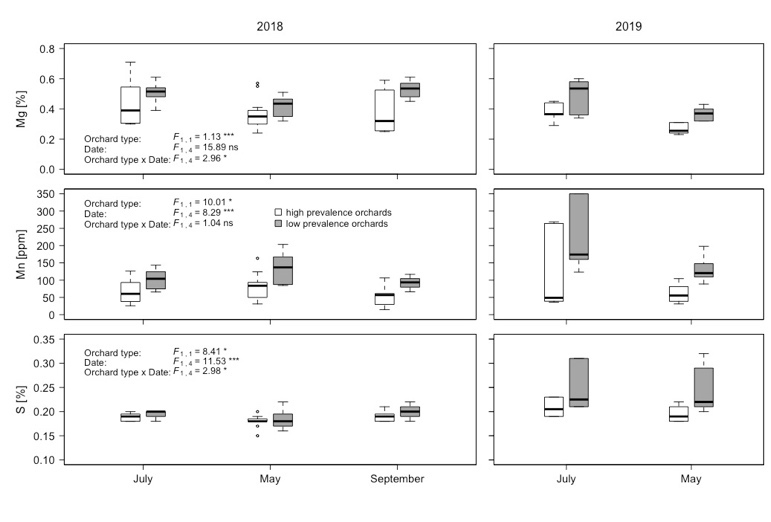


**Supplementary Figure 7:** Leaf nutrient concentration I. `Golden Delicious´ leaves were analysed for their chemical composition (Magnesium: Mg, Manganese: Mn, Sulphur: S) during the years 2018 and 2019 of different orchards (2018: n=8, 2019: n=6) in South Tyrol, Italy. We selected “orchard pairs” each managed by the same farmer but differing strongly in every year Alternaria leaf blotch and fruit spot infestation: high (white) and low (grey) prevalence orchards. See **Supplementary Table 6** for statistical analysis.


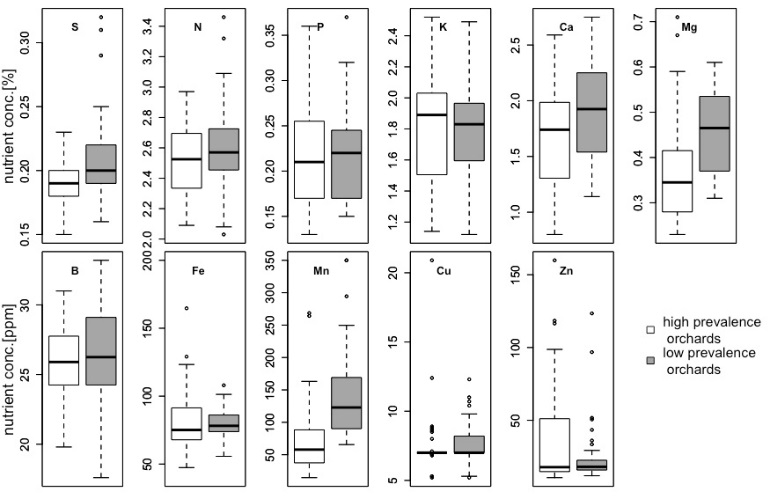


**Supplementary Figure 8:** Leaf nutrient concentration II. `Golden Delicious´ leaves were analysed for their chemical composition during the years 2018 and 2019 of different orchards (2018: n=8, 2019: n=6) in South Tyrol, Italy. We selected “orchard pairs” each managed by the same farmer but differing strongly in every year Alternaria leaf blotch and fruit spot attack: high (white) and low (grey) prevalence orchards. Except for Mg, Mn and S, “orchard type” and its interactions term with “Date” was never statistically significant. See **Supplementary Figure** **7** and **Supplementary Table 6** for statistical analysis.


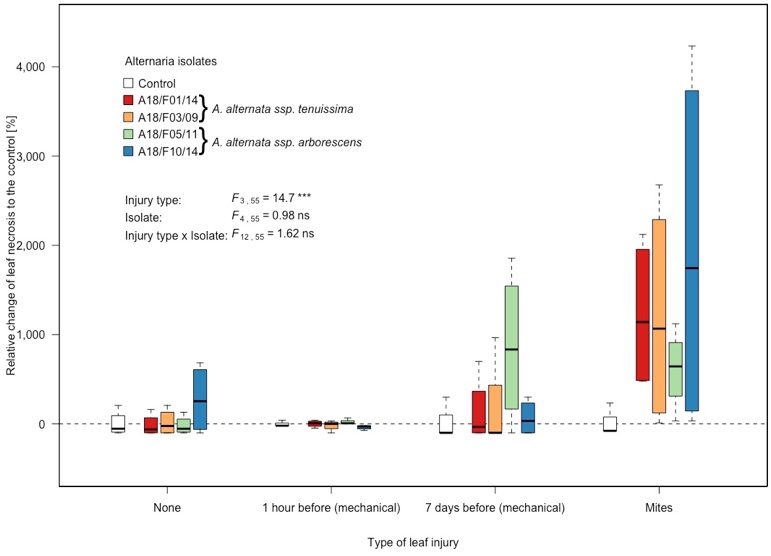


**Supplementary Figure 9:** Relative change of leaf necrosis in a leaf disc assay. The changes were in response to three types of leaf injury and four *Alternaria* isolates and are expressed relative to the corresponding control 12 days after inoculation. Type of leaf injuries are: none = healthy tissue, 1 hour before = injured with a pair of combination pliers 1 hour before inoculation, 7 days before= injured in the orchard with a pair of combination pliers 7 days before inoculation, Mites = leaves collected on the day of inoculation (29^th^ May 2019) from an orchard that was attacked severely by European red mites (*Panonychus ulmi*).


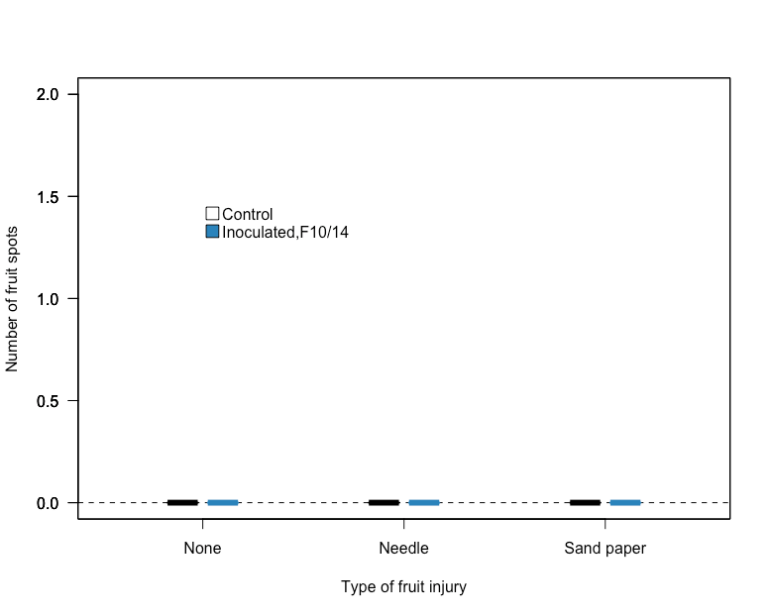


**Supplementary Figure 10:** Fruit spot formation after inoculation. The number of formed fruits spots was counted in response to two types of fruit injury and one Alternaria isolate (n = 4). Type of leaf injuries are: None = healthy tissue, Needle = injured with syringe needle (4 times per replicate) immediately before inoculation, Sand paper = injured with sand paper (~6 cm^2^) immediately before inoculation. After inoculation, apples were stored for 12 days in a climate chamber (25°C, 65% RH).


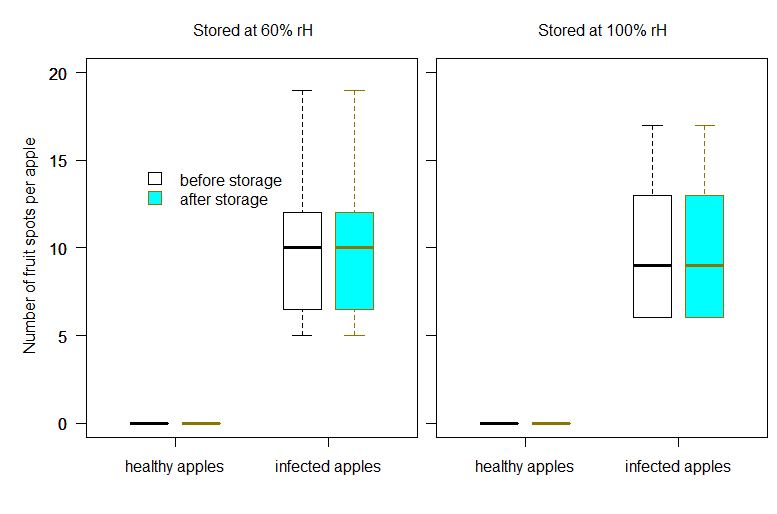


**Supplementary Figure 11:** Change in number of Alternaria fruit spots during storage: Apples were numbered for identification, fruits spots counted before (“before storage”) healthy and infected fruits were mixed and stored together in plastic boxes at room temperature (15-20°C) at 65% (healthy n=10; infected n=11) or 100% (healthy n=15; infected n=10) relative humidity (rH), respectively. After two months apples were counted again for fruit spots (“after storage”).


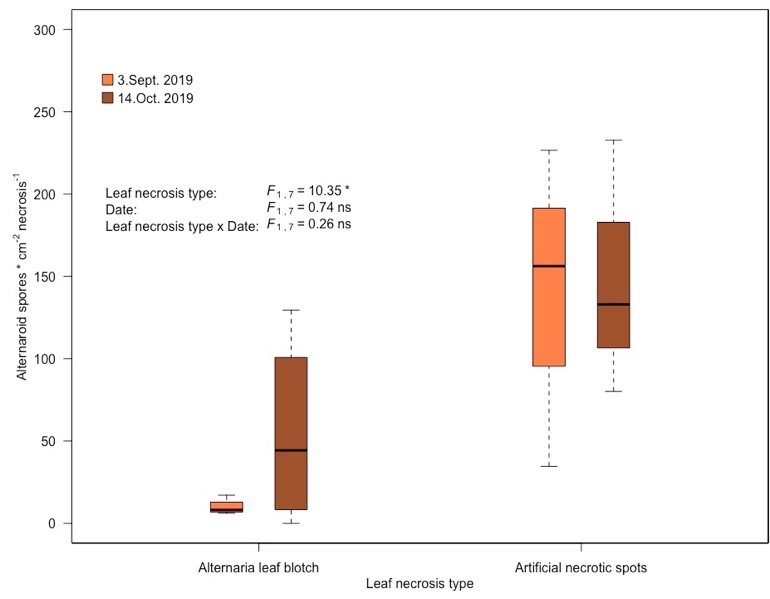


**Supplementary Figure 12:** Spore density of alternariod (Alternaria-like) spores washed from two types of leaf necrosis of `Golden Delicious´ trees: I) “Alternaria” leaf blotch, which was naturally occurred during summer; II) artificial necrotic spots, which were provoked by means of an herbicide (Carfentrazone-ethyl), in July 2019. Leaves were collected and subsequently analysed at two points in time from different trees within a randomized block design (n=4, each 5-6 leaves). Washed spores were counted with a haemocytometer and rescaled to the corresponding necrotic area. Spore density on artificial necrotic spots is significantly higher that on Alternaria leaf blotch (see **Supplementary Table 8**).


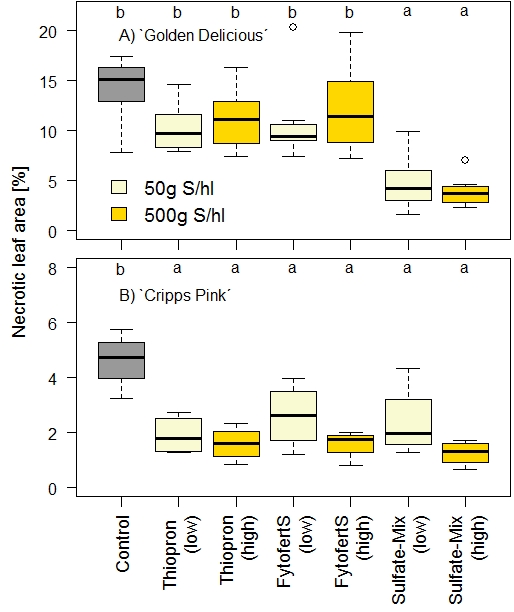


**Supplementary Figure 13**: Alternaria-associated leaf blotch controlled by different Sulphur-based leaf fertilizers (SBF). The three fertilizers (Thiopron®, Fytofert® S, Sulfate-Mix) were tested in two different concentrations, all corrected to 50g sulphur hl^-1^ (low, light yellow) and 500g sulphur hl^-1^ (high, dark yellow). Fertilizers were tested in the two apple varieties `Golden Delicious´ (A) and `Cripps Pink´ (Pink Lady®; B). The field trail was performed in a randomized block design (n = 4 per treatment) 2017 at Laimburg Research Center, South Tyrol, Italy.


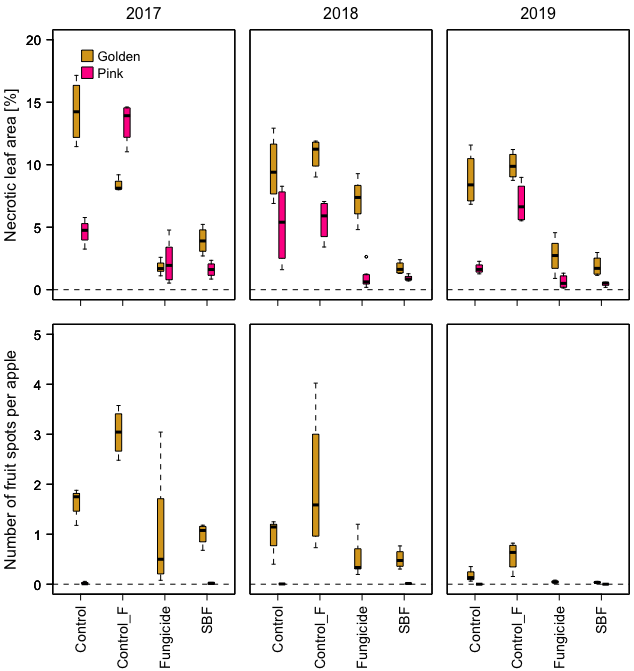


**Supplementary Figure 14**: Alternaria-associated leaf blotch and fruit spot controlled by fungicides and Sulphur-based leaf fertilizers (SBF). Treatments were tested in the two apple varieties `Golden Delicious´ (orange; fertilizer: Sulfate-Mix) and `Cripps Pink´ (Pink Lady®; pink; fertilizer: microfine sulphur) and compared to conventional practise (control; see also **Fig. 1**). The fungicides were tested in a separate long-term-experiment within the same orchard and had its own controls (control_F). The field trails were performed in a randomized block design (n = 4 per treatment) from 2017 to 2019 at Laimburg Research Center, South Tyrol, Italy.

**
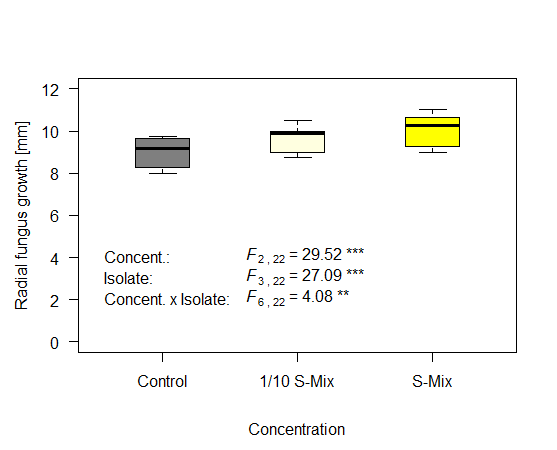

Supplementary Figure 15**: Fungicidal activity assay of the sulfate-mix (S-mix). Mycelia growth within 12 h of 4 different *Alternaria spp*. isolates (A02/F172, A02/F152, A15/271, A15/31) on control (PDA), on S-Mix (PDA+S-Mix in field-applied concentration) and on 1/10 S-Mix (PDA + 1/10 of field-applied concentration). The assay was performed on microtiter plates with 3 replicates per concentration and isolate.

**Supplementary Table 1: Composition and application of leaf fertilizers to control leaf blotch for `Golden Delicious´ and `Cripps Pink´.**

| Variety | **Golden Delicious** | **Cripps Pink (Pink Lady^®^)** | |
| --- | --- | --- | --- |
| Agent name^1^ | Sulfate-Mix | Thiopron®  (2018) | Thiovit®  (2019) |
| Composition/ Dosage  (for 15 hl/ha) | Magnesium sulfate, 3780 g/hl  (K&S EPSO TOP 16/13)  Manganese sulfate, 33 g/hl  (Gobbi Mangan 32 PG)  Zink sulfate,115 ml/hl  (Gobbi Zink LG) | 0.242 l/hl = 200 g S/hl | 240g/hl =  190g S/hl |
| Timing | Starting in June, ~every 3rd week, ideally treatment after heavy rainfalls | Starting in July, ~every 3rd week, ideally treatment after heavy rainfalls | |
| Frequency treatment | 4-5 per season | 5-6 per season | |
| Last treatment | End of August/ Begin of September | Mid of October | |

^1^This variety-specific treatment is based on a pre-experiment, where we found that multiple sulphur-types had an effect on `Cripps Pink´, while `Golden Delicious´ was most affected in the presence of all the three mentioned sulfates (**Supplementary Fig. 13**).

^2^ Estimated prices, which can strongly vary.

**Supplementary Table** **2: Statistical analyses of the effect of treatment on Alternaria leaf blotch on apple leaves.**

| Variety | Factor | numDf | denDF | F-Value | P-value |
| --- | --- | --- | --- | --- | --- |
|  |  |  |  |  |  |
|  | Sulphur-based leaf fertilizers | | | |  |
| `Golden Delicious´ | (Intercept) | 1 | 39 | 350.448 | **<.0001** |
|  | Year | 2 | 39 | 9.415 | **0.0005** |
|  | Treatment | 1 | 39 | 185.147 | **<.0001** |
|  | Year:Treatment | 2 | 39 | 2.718 | 0.0785 |
| `Cripps Pink´ | (Intercept) | 1 | 37 | 36.381 | **<.0001** |
|  | Year | 2 | 37 | 5.958 | **0.0057** |
|  | Treatment | 1 | 37 | 28.722 | **<.0001** |
|  | Year:Treatment | 2 | 37 | 2.108 | 0.136 |
|  |  |  |  |  |  |
|  | Fungicides |  |  |  |  |
| `Golden Delicious´ | (Intercept) | 1 | 27 | 921.050 | **<.0001** |
|  | Year | 2 | 27 | 47.944 | **<.0001** |
|  | Treatment | 1 | 27 | 202.191 | **<.0001** |
|  | Year:Treatment | 2 | 27 | 7.246 | **0.003** |
| `Cripps Pink´ | (Intercept) | 1 | 27 | 229.629 | **<.0001** |
|  | Year | 2 | 27 | 18.897 | **<.0001** |
|  | Treatment | 1 | 27 | 295.179 | **<.0001** |
|  | Year:Treatment | 2 | 27 | 20.792 | **<.0001** |

Effects were tested by a *F* test from linear mixed-effects model (LMM) analyses for the two apple varieties (`Golden Delicious´ and `Cripps Pink´). “mean necrotic leaf area” was set as response variable with treatment (Sulphur-based fertilizers vs. control or Fungicides vs. control) and “Year” (2017-2019) as fixed effects. “Year” and “Block” (Replicate; n = 4) were specified as random effects with separate slopes (Year | Block). Significant differences at *P* ≤ 0.05 set in bold.

**Supplementary Table 3: Effect of treatment on the reduction of Alternaria fruit spot on apples.**

| Variety | Factor | numDf | denDF | F-Value | P-value |
| --- | --- | --- | --- | --- | --- |
| `Golden Delicious´ | SBF |  |  |  |  |
|  | (Intercept) | 1 | 53 | 111.338 | **<.0001** |
|  | Year | 2 | 53 | 88.632 | **<.0001** |
|  | Treatment | 1 | 53 | 45.944 | **<.0001** |
|  | Year:Treatment | 2 | 53 | 4.103 | **0.022** |
|  |  |  |  |  |  |
|  | Fungicides |  |  |  |  |
|  | (Intercept) | 1 | 27 | 12.929 | **0.001** |
|  | Year | 2 | 27 | 19.455 | **<.0001** |
|  | Treatment | 1 | 27 | 43.995 | **<.0001** |
|  | Year:Treatment | 2 | 27 | 4.845 | **0.016** |
| `Cripps Pink´ | SBF |  |  |  |  |
|  | (Intercept) | 1 | 37 | 4.796 | 0.0359 |
|  | Year | 2 | 37 | 1.499 | 0.237 |
|  | Treatment | 1 | 37 | 0.129 | 0.721 |
|  | Year:Treatment | 2 | 37 | 0.402 | 0.672 |
|  |  |  |  |  |  |

Effects were tested by a *F* test referring to the linear mixed-effects model (LMM) for the apple variety `Golden Delicious´ and `Cripps Pink´, respectively. Fixed effects were treatment (Sulphur-based fertilizers vs. control or Fungicides vs. control, only available for `Golden Delicious´) and Year (2017-2019). Year and Plot (n = 4) was specified as random effect. Significant differences at *P* ≤ 0.05 set in bold.

**Supplementary Table 4: Pearson correlation between Alternaria leaf blotch and fruit spots.**

|  | All data | | | Only Sulphate-Mix and control | | |
| --- | --- | --- | --- | --- | --- | --- |
|  | r | d.f. | P-value | r | d.f. | P-value |
| 2017 | 0.712 | 102 | <0.001 | 0.701 | 22 | <0.001 |
| 2018 | 0.735 | 73 | <0.001 | 0.780 | 21 | <0.001 |
| 2019 | 0.375 | 93 | <0.001 | 0.535 | 21 | 0.008 |

The year-wise analysis is based on our field trials for the apple variety `Golden Delicious´. “All data” includes leaf blotch and fruits spot data of all treatments tested (2017: 13, 2018: 10, 2019: 12 treatments; n=4), while “Sulphate-Mix and control” include only data of the two corresponding treatments. Generally, for every plot (replicate), both tree sides (east and west) were evaluated separately.

**Supplementary Table 5: Results of linear mixed model analyses of leaf nutrient content on leaf blotch area.**

| Model: | mod1: PCA based | | |  |  | mod2: Including S | | | mod3: Including Mg | | | mod2a (mod2 excluding B) | | |
| --- | --- | --- | --- | --- | --- | --- | --- | --- | --- | --- | --- | --- | --- | --- |
| AIC | 394.35 |  |  |  |  | 393.31 | | | 409.46 | | | 393.12 | | |
| R^2^_marginal_ | 48.5 |  |  |  |  | 48.4 | | | 43.8 | | | 46.9 | | |
| R^2^_conditional_ | 96.3 |  |  |  |  | 96.2 | | | 94.7 | | | 96.1 | | |
|  |  |  |  |  |  |  | | |  | | |  |  |  |
| Terms | d.f. | F | P |  | Terms | d.f. | F | P | d.f. | F | P | d.f. | F | P |
| Variety | 1,101 | 0.79 | 0.3774 |  | Variety | 1,101 | 1.99 | 0.1616 | 1,101 | 2.04 | 0.1558 | 1,101 | 1.07 | 0.3026 |
| **PCA 1 (90.1%**  **of S & Mg)** | **1,77** | **17.19** | **0.0001** |  | **S** | **1,79** | **17.00** | **0.0001** | - | - | - | **1,81** | **16.71** | **0.0001** |
| **PCA 2** | **1,77** | **5.22** | **0.0251** |  | **Mg** | - | - | - | **1,79** | **12.08** | **0.0008** | - | - | - |
| **Mn** | **1,77** | **5.28** | **0.0243** |  | **Mn** | **1,79** | **5.49** | **0.0216** | **1,79** | **7.94** | **0.0061** | **1,81** | **13.26** | **0.0005** |
| **P** | **1,77** | **4.74** | **0.0325** |  | **P** | **1,79** | **4.57** | **0.0357** | 1,79 | 3.49 | 0.0656 | 1,81 | 3.95 | 0.0503 |
| B | 1,77 | 2.96 | 0.0888 |  | B | 1,79 | 2.44 | 0.1226 | 1,79 | 0.20 | 0.6568 | - | - | - |
| Variety x PCA 1 | 1,77 | 2.80 | 0.0985 |  | Variety x S | 1,79 | 1.56 | 0.2155 | **-** | **-** | **-** | 1,81 | 0.32 | 0.5707 |
| Variety x PCA 2 | 1,77 | 0.55 | 0.4603 |  | Variety x Mg | - | - | - | 1,79 | 2.46 | 0.1206 | - | - | - |
| **Variety x Mn** | **1,77** | **4.66** | **0.0341** |  | **Variety x Mn** | **1,79** | **5.04** | **0.0276** | 1,79 | 2.31 | 0.1323 | **1,81** | **7.81** | **0.0065** |
| Variety x P | 1,77 | 1.80 | 0.1840 |  | Variety x P | 1,79 | 1.65 | 0.2031 | 1,79 | 0.97 | 0.3265 | 1,81 | 1.15 | 0.2875 |
| Variety x B | 1,77 | 0.60 | 0.4427 |  | Variety x B | 1,79 | 0.96 | 0.3293 | 1,79 | 0.9220 | 0.3674 | - | - | - |

Significant (p < 0.05) model terms are highlighted in bold.

**Supplementary Table 6: Effect of “Orchard type” and sampling “Date” of different `Golden Delicious´ orchards on Magnesium (Mg), Manganese (Mn) and Sulphur (S) concentration in leaves of apple trees.**

| Response variable | Factor | numDf | denDF | F-Value | P-value |
| --- | --- | --- | --- | --- | --- |
| Mg | (Intercept) | 1 | 64 | 140.947 | <.0001 |
|  | Date | 4 | 64 | 15.892 | **<.0001** |
|  | Orchard type | 1 | 6 | 1.131 | 0.329 |
|  | Date:Orchard type | 4 | 64 | 2.955 | **0.026** |
| Mn | (Intercept) | 1 | 64 | 135.553 | <.0001 |
|  | Date | 4 | 64 | 8.288 | **<.0001** |
|  | Orchard type | 1 | 6 | 10.013 | **0.020** |
|  | Date:Orchard type | 4 | 64 | 1.040 | 0.394 |
| S | (Intercept) | 1 | 64 | 4498.162 | <.0001 |
|  | Date | 4 | 64 | 11.527 | **<.0001** |
|  | Orchard type | 1 | 6 | 8.407 | **0.027** |
|  | Date:Orchard type | 4 | 64 | 2.987 | **0.025** |
| N | (Intercept) | 1 | 64 | 1267.069 | <.0001 |
|  | Date | 4 | 64 | 51.971 | **<.0001** |
|  | Orchard type | 1 | 6 | 0.190 | 0.678 |
|  | Date:Orchard type | 4 | 64 | 1.619 | 0.180 |
| P | (Intercept) | 1 | 64 | 153.401 | <.0001 |
|  | Date | 4 | 64 | 4.507 | **0.0029** |
|  | Orchard type | 1 | 6 | 0.030 | 0.869 |
|  | Date:Orchard type | 4 | 64 | 0.433 | 0.784 |
| K | (Intercept) | 1 | 64 | 1144.869 | <.0001 |
|  | Date | 4 | 64 | 22.900 | **<.0001** |
|  | Orchard type | 1 | 6 | 0.002 | 0.963 |
|  | Date:Orchard type | 4 | 64 | 1.693 | 0.163 |
| Ca | (Intercept) | 1 | 64 | 1096.730 | <.0001 |
|  | Date | 4 | 64 | 57.408 | **<.0001** |
|  | Orchard type | 1 | 6 | 3.096 | 0.129 |
|  | Date:Orchard type | 4 | 64 | 0.750 | 0.562 |
| B | (Intercept) | 1 | 64 | 2923.533 | <.0001 |
|  | Date | 4 | 64 | 5.374 | **0.0009** |
|  | Orchard type | 1 | 6 | 0.443 | 0.530 |
|  | Date:Orchard type | 4 | 64 | 0.715 | 0.585 |
| Fe | (Intercept) | 1 | 64 | 1000.471 | <.0001 |
|  | Date | 4 | 64 | 6.876 | **0.0001** |
|  | Orchard type | 1 | 6 | 0.398 | 0.551 |
|  | Date:Orchard type | 4 | 64 | 1.520 | 0.207 |
| Cu | (Intercept) | 1 | 64 | n.a.* | n.a. |
|  | Date | 4 | 64 | n.a. | n.a. |
|  | Orchard type | 1 | 6 | n.a. | n.a. |
|  | Date:Orchard type | 4 | 64 | n.a. | n.a. |
| Zn | (Intercept) | 1 | 64 | 16.476 | 0.0001 |
|  | Date | 4 | 64 | 1.1063 | 0.361 |
|  | Orchard type | 1 | 6 | 0.815 | 0.401 |
|  | Date:Orchard type | 4 | 64 | 1.610 | 0.183 |

Effects were tested by a *F* test referring to the linear mixed-effects model (LMM) for each of the nutrients separately as a response variable. “Date” and “Orchard type” (high vs. low prevalence) their interaction were included as fixed effects. “Date” (n=5, with 2 replicates each per orchard) and “Orchard ID” (2018: n = 8; 2019: n=6) were specified as random effect with separate slopes (Date|Orchard ID). Significant differences at *P* ≤ 0.05 are set in bold.
* No statistical analysis was possible for Cu as there was almost no variation in the data (almost cases all <7 ppm) and the LMM did not converge.

**Supplementary Table 7: Effect of injury type and *Alternaria spp.* isolates on the occurrence and size of leaf necrosis at a `Golden Delicious´ leaf disc assay**

| Variety | Factor | numDf | denDF | F-Value | P-value |
| --- | --- | --- | --- | --- | --- |
| `Golden Delicious´ | (Intercept) | 1 | 55 | 85.375 | **<.001** |
|  | Injury type | 3 | 55 | 14.702 | **<.001** |
|  | Isolate | 4 | 55 | 0.983 | 0.424 |
|  | Injury type:Isolate | 12 | 55 | 1.624 | 0.112 |

Effects were tested by a *F* test referring to the linear mixed-effects model (LMM) for leaf necrosis area as depended variable. Fixed effects were “Injury type” (n = 4) and “Isolate” (n = 4). „Replicate” (n = 4) was specified as random effect. Significant differences at p ≤ 0.05 set in bold.

**Supplementary Table 8: Effect of leaf necrosis type of `Golden Delicious´ leaves on the spore density of Alternaroid spores.**

| Variety | Factor | numDf | denDF | F-Value | P-value |
| --- | --- | --- | --- | --- | --- |
| `Golden Delicious´ | (Intercept) | 1 | 7 | 21.738 | 0.002 |
|  | Leaf necrosis type | 1 | 7 | 10.353 | **0.015** |
|  | Date | 1 | 7 | 0.741 | 0.418 |
|  | Leaf necrosis type:Date | 1 | 7 | 0.257 | 0.628 |

Artificially necrosis was provoked by means of a herbicide (Carfentrazone-ethyl) and spore density was compared after almost 2 month to naturally occurring “Alternaria” leaf blotch. Effects were tested by a *F* test referring to the linear mixed-effects model (LMM) for spore density of `Golden Delicious´ leaves. Fixed effects were „Leaf necrosis type” (artificially provoked vs. “Alternaria” leaf blotch) and “Date” of leaf collection (3. Sept. 2019 and 14. Oct. 2019). “Replicate” (n = 4) was specified as random effect. Significant differences at p ≤ 0.05 set in bold.
